# The strength of interspecies interaction in a microbial community determines its susceptibility to invasion

**DOI:** 10.1371/journal.pbio.3002889

**Published:** 2024-11-07

**Authors:** Suraya Muzafar, Ramith R. Nair, Dan I. Andersson, Omar M. Warsi

**Affiliations:** Dept. of Medical Biochemistry and Microbiology, Uppsala University, Uppsala, Sweden; Universität Bern: Universitat Bern, SWITZERLAND

## Abstract

Previous work shows that a host’s resident microbial community can provide resistance against an invading pathogen. However, this community is continuously changing over time due to adaptive mutations, and how these changes affect the invasion resistance of these communities remains poorly understood. To address this knowledge gap, we used an experimental evolution approach in synthetic communities of *Escherichia coli* and *Salmonella* Typhimurium to investigate how the invasion resistance of this community against a bacterium expressing a virulent phenotype, i.e., colicin secretion, changes over time. We show that evolved communities accumulate mutations in genes involved in carbon metabolism and motility, while simultaneously becoming less resistant to invasion. By investigating two-species competitions and generating a three-species competition model, we show that this outcome is dependent on the strength of interspecies interactions. Our study demonstrates how adaptive changes in microbial communities can make them more prone to the detrimental effects of an invading species.

## Abstract 2

Microbial communities are constantly evolving, affecting their ability to resist invading pathogens. This study shows that adaptive changes in resource competition between species can make bacterial communities more susceptible to invasion.

## Introduction

An important factor determining the outcome of host-pathogen interactions is the interaction between the host’s residential microbial community and the invading pathogen. Several studies investigating these interactions in different host species have shown them to either protect against [1–5] or facilitate a pathogen’s invasion [6,7]. Mechanisms that protect the host include resource competition, colonization resistance, and modulation of the host’s immune response, while mechanisms that facilitate invasion include the secretion of metabolites that can allow the pathogen to initiate infections in the host. Building on this knowledge, research is also being conducted to identify and characterize synthetic microbial communities that could protect a host against specific pathogens [8–10], which potentially could be utilized in precision medicine and therapeutics. Interestingly, the outcome of these interactions is not only dictated by the identity of the species present in these communities but also by other factors such as the secretion of specific molecules or species abundances [11,12]. Given the importance of these interactions to a host’s health, it is thus important to determine the underlying mechanisms, both physiological and ecological, that can affect the outcome of the competition between the host’s residential microbial community and a pathogen.

To overcome the invasion resistance posed by the host’s residential microbial community, several pathogens have been shown to secrete various toxins. For example, using bacterial genome-wide association studies and high-throughput phenotyping, it was suggested that the enteric pathogen *Shigella sonnei* outcompetes the gut commensal *Escherichia coli* by secreting colicins, which are proteinaceous toxins that specifically target *E*. *coli* strains [13]. The secretion of such toxins has been hypothesized to result in the epidemiological success of this pathogen. Similarly, an analysis of 1,181 *E*. *coli* strains of fecal origin showed a strong positive correlation between bacteriocin-producing genes and virulence factors, suggesting the role of these toxins during the pathogen invasion [14]. This correlation was specifically observed for ExPEc (extraintestinal) pathogenic *E*. *coli* strains. In another study [15], *Salmonella* Dublin, which is a cattle-adapted pathogen, was shown to carry 2 different Type VI secretion systems that were involved in interbacterial competition. These studies have given us valuable insights into how pathogens can dominate in competition against the host’s residential microbial community by secreting specific toxins.

On the other hand, the host’s resident microbial community is also continuously changing with several studies demonstrating adaptive evolution in these communities [16,17]. These studies have demonstrated how these evolutionary changes can be observed over different time scales [18] and can affect important resource utilization pathways [19]. Importantly, these adaptive changes can in turn affect the community’s resistance against an invading microbial species. Thus, increased productivity of a microbial community, either through faster growth rates or increased cell density of its community members, results in a lower likelihood of invasion by a microbial species due to lower nutrient availability for the invader [20]. Another study showed that the joint evolution of microbial communities growing in a toxic medium increased invasion resistance [21]. Although the mechanism underlying this is unclear, the authors hypothesized that this observation could either be due to resource specialization through niche-partitioning or due to increased productivity of the evolved community members, both of which would result in fewer nutrients for the invading microbial species.

Although these studies provide an important framework to investigate the relationship between an evolving community and invasion by a microbial species, whether or not these rules are applicable during an invasion by a toxin-secreting pathogen remains an outstanding question. To answer this question systematically, we employed an experimental evolution approach in synthetic microbial communities and determined how adaptive changes in these communities affect the invasion by a toxin-secreting bacteria. Specifically, we determined the rate of invasion of a colicin-producing natural isolate of *E*. *coli* (ECOR 11, henceforth designated as colicin-producing *E*. *coli*) in ancestral and evolved synthetic two-species communities of *Escherichia coli* (henceforth mentioned as *E*. *coli*) and *Salmonella enterica* serovar Typhimurium LT2 (henceforth designated as *S*. Typhimurium). Colicins are a commonly occurring class of proteinaceous polymorphic toxins that specifically target *E*. *coli* [22]. Importantly, colicin secretion by the pathogen has been shown to contribute to its success during an invasion [13,14,23]. This setup allowed us to mechanistically link the success of an invasion due to a virulent phenotype to adaptive changes in a microbial community.

Our results demonstrate strong eco-evolutionary dynamics influencing the success of the colicin-producing *E*. *coli* strain’s invasion, where we observed a higher rate of invasion in evolved communities as compared to the ancestral communities. Two-species competition experiments and whole-genome sequencing analysis of evolved clones demonstrate that this outcome was observed due to increased resource utilization and ecological specialization in the evolved communities.

## Results

### Ancestral *E*. *coli* and *S*. Typhimurium strains form a community with a single steady state

**Fig 1:**
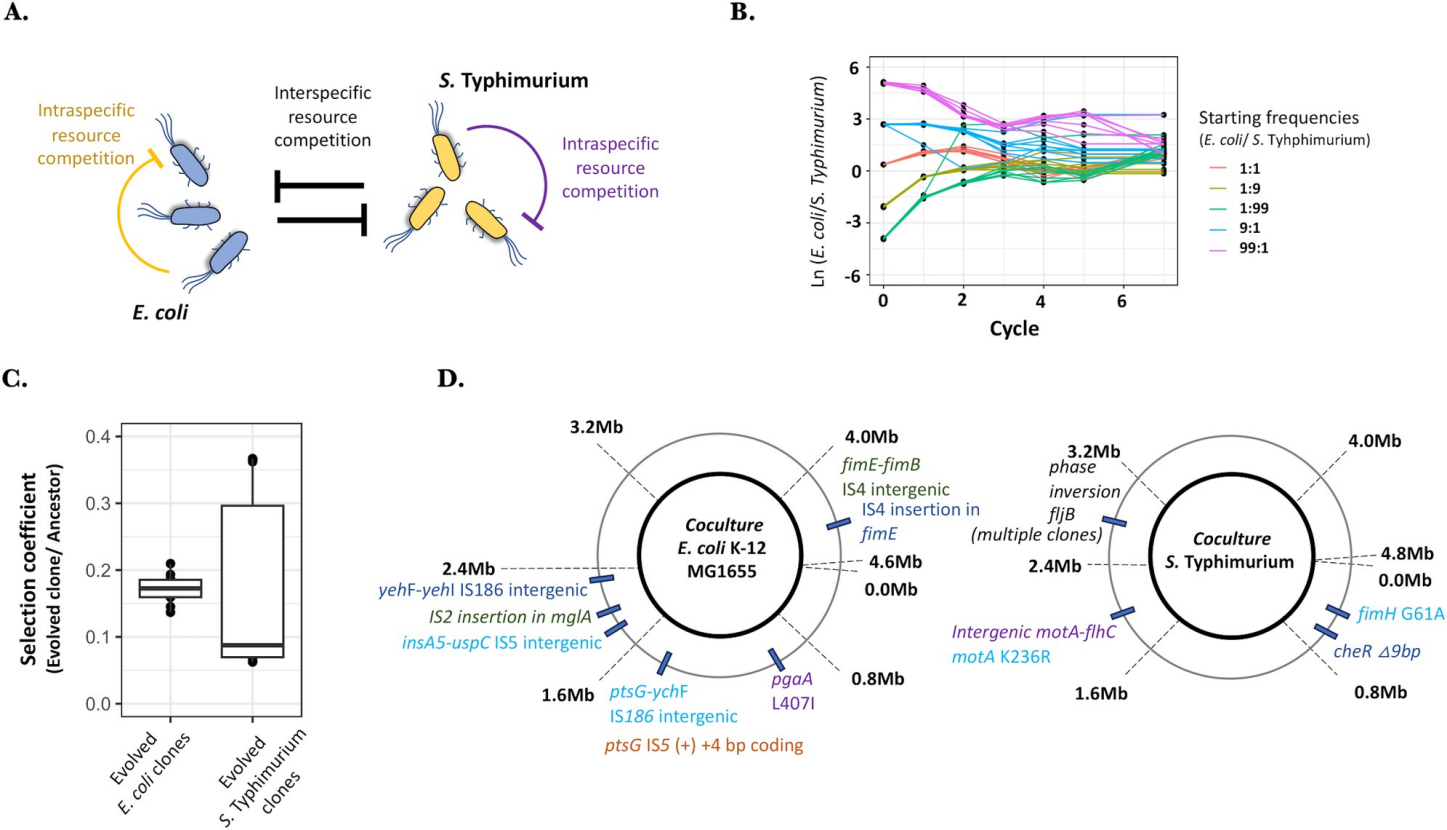
Evolved communities of *E*. *coli* and *S*. Typhimurium are more efficient in resource utilization. (A) A depiction of the two-species synthetic microbial community of *E*. *coli* and *S*. Typhimurium used in the study. There is significant resource usage overlap between *E*. *coli* and *S*. Typhimurium in nutrient-rich LB media, suggesting strong interspecies and intraspecies resource competition. (B) Coexistence and steady state were determined in the synthetic two-species community by performing invasion experiments using different starting frequencies of *E*. *coli*: *S*. Typhimurium (ranging from 1:99 to 99:1). Eight replicates were used in each case. The Y-axis represents natural log-transformed values for the ratios between *E*. *coli* and *S*. Typhimurium. The data underlying this figure can be found in S1 Data, in the sheet titled Fig 1B. (C) Clones of *E*. *coli* and *S*. Typhimurium that were isolated from the evolved communities showed increased fitness when competed against the respective ancestors. The Y-axis represents the selection coefficient calculated by competing the evolved clones with the respective ancestral strains. Four replicates were used in each case, and the line in the center represents the median value. The data underlying this figure can be found in S1 Data, in the sheet titled Fig 1C. (D) Mutations identified by performing whole-genome sequencing analysis of evolved *E*. *coli* and *S*. Typhimurium clones. Five clones isolated from different communities were whole-genome sequenced for each species. Different mutations occurring in the same clone are denoted by the same color of the gene label.

The synthetic two-species microbial community used in our study consists of *E*. *coli* MG1655 and *S*. Typhimurium LT2 grown in nutrient-rich lysogeny broth (LB, Fig 1A). We started communities with a set of different frequencies of *E*. *coli*: *S*. Typhimurium that ranged between ~1:99 and 99:1 (Fig 1B) with 8 replicates for each ratio. These communities were passaged for 7 cycles of growth (~56 generations), and all of these converged to an average ratio of 3:1 (*E*. *coli* / *S*. Typhimurium ratios ranging from 1.8 ± 0.79 to 4.21 ± 1.59, Fig 1B). This result indicates that these 2 microbial species reach a steady state in nutrient-rich LB. To determine the extent of overlap in resource usage in this medium between *E*. *coli* and *S*. Typhimurium, single-species growth was measured in spent medium obtained from overnight growth of the other species. While *E*. *coli* did not grow on the spent medium obtained after the overnight growth of *S*. Typhimurium, the latter showed minimal growth on the spent medium obtained after the overnight growth of *E*. *coli* (final OD_600_ = 0.203 ± 0.004, S1A Fig). Furthermore, restocking the spent medium with nutrients resulted in both the species growing similar to those obtained in fresh medium (S1B Fig). Thus, the interaction between *E*. *coli* and *S*. Typhimurium is primarily driven by competition for resources and not through the production of inhibitory molecules. This indicates the predominance of scramble-type competition between these 2 species in LB.

### Evolved clones of *E*. *coli* and *S*. Typhimurium show increased fitness and contain mutations in genes involved in carbon metabolism, adhesion, and motility

To generate evolved communities, we passaged ten independent co-culture communities of *E*. *coli* and S. Typhimurium for 55 transfers (approximately 440 generations). In all 10 communities, both *E*. *coli* and S. Typhimurium coexisted throughout the experiment. At the end-point of this experiment, we isolated the evolved clones and determined their fitness relative to the respective ancestral strains by performing competition experiments (described in Materials and methods). For each evolved clone, we observed an increase in relative fitness that ranged from 0.136 ± 008 gen^-1^ to 0.209 ± 0.025 gen^-1^ for evolved *E*. *coli* clones, and 0.062 ± 0.006 gen^-1^ to 0.36 ± 0.01 gen^-1^ for evolved *S*. Typhimurium clones (Fig 1C, significantly different from respective controls at *p* < 0.05 for a Student’s *t* test with Bonferroni correction applied for multiple comparisons, S1 Table). We next investigated if the increased relative fitness of these evolved clones was due to an increase in exponential growth rate or an increased growth yield (i.e., higher stationary phase density). Individual growth curves of the evolved clones were compared to those of the ancestral strains. For *E*. *coli*, 6 out of 10 evolved clones had a higher stationary density as compared to the ancestral *E*. *coli*, while only 1 evolved clone had a higher exponential growth rate compared to the ancestral strain (*p* < 0.05 for a Student’s *t* test, with Bonferroni correction applied for multiple comparisons, S2 Table). For *S*. Typhimurium, 2 out of 10 evolved clones had a higher stationary density than the ancestral strain while the exponential growth rates were not different between any of the evolved clones and the ancestral strains (*p* < 0.05 for a Student’s *t* test, with Bonferroni correction applied for multiple comparisons, S2 Table). Thus, in at least 8 out of 20 evolved clones, the increase in fitness can be attributed to increased stationary phase growth density (i.e., growth yield).

In the ancestral community, *E*. *coli* and *S*. Typhimurium coexisted and reached a final average ratio of approximately 3:1 (*E*. *coli*: *S*. Typhimurium). To determine if this ratio changed for the evolved clones, we grew cognate pairs of *E*. *coli* and *S*. Typhimurium (isolated from the same community) together and measured their final ratios, relative to the ratios of the ancestral strains (same set of evolved *E*. *coli* and *S*. Typhimurium clones are used throughout the study). The relative ratios for the evolved communities varied between 0.53 ± 0.02 and 8.12 ± 2.3, with the ratios of 4 evolved communities being significantly different from that of the ancestral community (statistically significant at *p* < 0.05 for a Student’s *t* test with Bonferroni’s correction applied for multiple testing, S2 Fig).

Next, to determine changes in resource overlap between *E*. *coli* and *S*. Typhimurium in the ancestral and evolved communities, a comparison of stationary phase densities between the ancestral and evolved states in the appropriate spent medium was performed. Thus, the growth of the evolved *E*. *coli* and *S*. Typhimurium clones was measured in spent medium obtained after overnight growth of the cognate-evolved *S*. Typhimurium and *E*. *coli* clones, respectively. These results showed that the evolved *E*. *coli* clones grew to a higher population density in spent medium from the cognate-evolved *S*. Typhimurium clones as compared to the population density of the ancestral *E*. *coli* on spent medium of the ancestral *S*. Typhimurium (statistically significant for 6 out of the 10 evolved clones at *p* < 0.05 for a Student’s *t* test with Bonferroni’s correction applied for multiple testing, S3A Fig). On the other hand, evolved *S*. Typhimurium clones grew to a lower population density in the spent medium of the cognate evolved *E*. *coli* clones as compared to the population density of the ancestral *S*. Typhimurium on spent medium of ancestral *E*. *coli* (statistically significant for 5 out of the 10 evolved clones at *p* < 0.05 for a Student’s *t* test with Bonferroni’s correction applied for multiple testing, S3B Fig). Although these results indicate altered resource overlap for *E*. *coli* and *S*. Typhimurium in the evolved communities compared to the ancestral communities, more work is needed to understand how these changes affect resource usage abilities between the 2 species.

To identify the type of mutations that was present in these evolved clones, we whole genome sequenced 5 clones each of *E*. *coli* and *S*. Typhimurium (Fig 1D and S3 Table). In all the clones, we observed mutations in genes that were either involved in carbon metabolism, adhesion, or motility. Thus, among the *E*. *coli* strains, we observed mutations in genes *mglA* (methyl galactoside ABC transporter ATP-binding unit) and *ptsG* (glucose-specific phospotransferase system, observed in 2 out of the 5 clones), with both these genes coding for transporters for different sugars [24]. For both these genes, we observed loss of function mutations. One of the clones that contained a mutation in *ptsG* also showed a mutation in gene *uspC* (universal stress protein). Besides these mutations, we also observed mutations in the gene *fimE* (regulator of fimbrial structural genes) and *pgaA* (D-glucosamine export porin), with both these genes contributing to the adhesion phenotype in *E*. *coli* [24]. Importantly, loss of function mutations in the *fimE* gene has been previously shown to contribute to increased fitness in rich media [25]. In the evolved *S*. Typhimurium strains, we observed mutations in genes *motA* (flagellar motility protein), *fimH* (fimbriae minor protein), *cheR* (methyltransferase involved in chemotaxis), and *fljB* (flagellar protein), with all these genes encoding for proteins involved in motility and adhesion.

### Evolved communities are more prone to invasion by colicin-producing *E*. *coli*

**Fig 2:**
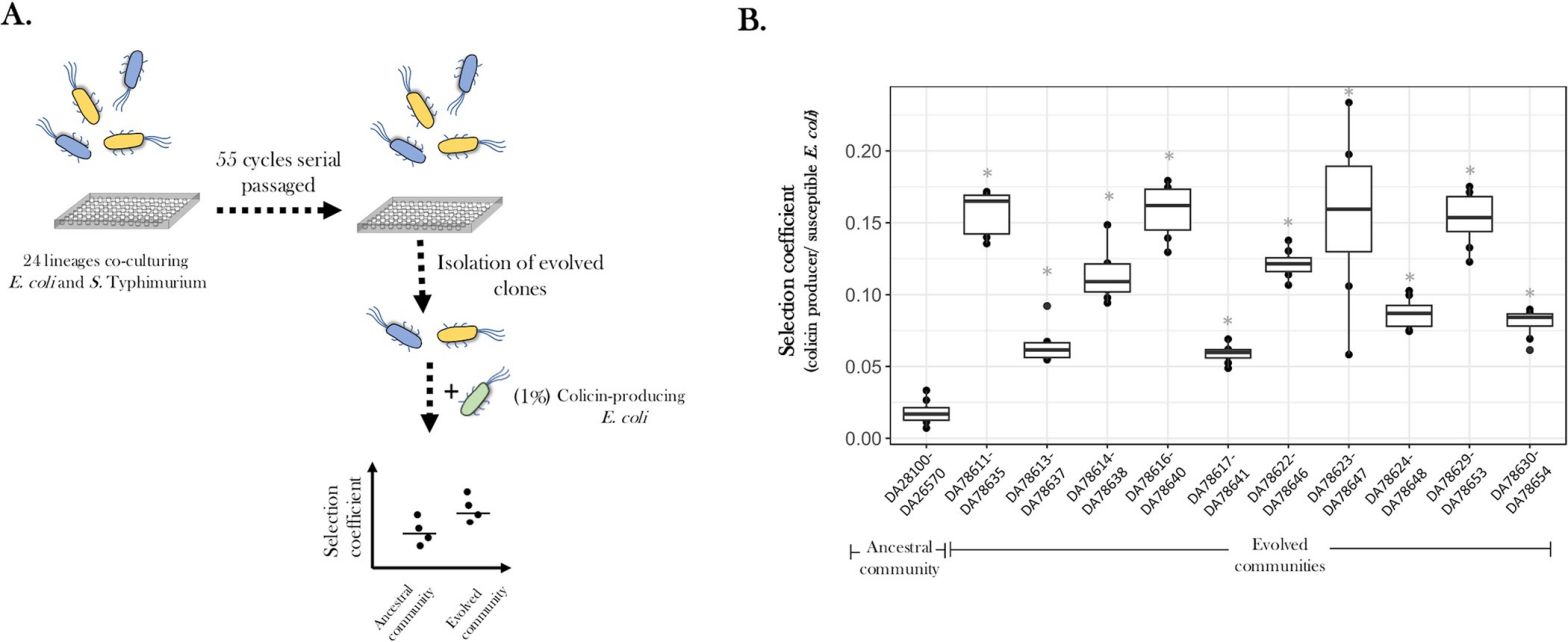
Evolved communities of *E*. *coli* and *S*. Typhimurium are less resistant to invasion. (A) Schematic representation of experiments performed to determine invasion resistance of ancestral and evolved communities of *E*. *coli* and *S*. Typhimurium against a colicin-producing *E*. *coli*. Evolved communities were formed by mixing 10 evolved *E*. *coli* and *S*. Typhimurium clones that were isolated from the same evolving community. (B) Invasion resistance was determined by measuring selection coefficients in competition experiments between colicin-producing *E*. *coli* and susceptible *E*. *coli* in ancestral and evolved communities. Eight replicates were used in each case and the line in the center represents the median value. The X-axis represents the ancestral and evolved communities and the Y-axis represents the selection coefficients showing the change in frequency of the colicin-producing *E*. *coli* with respect to the susceptible *E*. *coli*. A Student’s *t* test (with Bonferroni’s correction for multiple testing) was performed to determine statistically significant differences (denoted as * for *p* < 0.05) between the ancestral and each of the evolved states. The numbers on the X-axis represent the strain IDs for ancestral and evolved clones of *E*. *coli* and *S*. Typhimurium used in the study. The data underlying this figure can be found in S1 Data, in the sheet titled Fig 2B.

To investigate the effect of the adaptive evolution of a microbial community on its ability to resist invasion by a toxin-producing bacterium, we measured the rate of increase of a colicin-producing *E*. *coli* strain in both the ancestral and evolved communities when starting at a low frequency of approximately 1% of the population (Fig 2A). To control for the level of adaptive genetic diversity in the evolved communities, which could confound invasion resistance of these communities, we generated evolved communities by mixing cognate pairs of evolved clones from each community. Overall, the rate of increase in the frequency of the invading colicin-producing *E*. *coli* was higher in the evolved communities as compared to the ancestral communities (Fig 2B and S4 Table). Thus, the selection coefficient for the rate of increase of the colicin-producing *E*. *coli* over susceptible *E*. *coli* was 0.02 ± 0.008 gen^-1^ in the ancestral community, while it varied between 0.06 ± 0.006 gen^-1^ to 0.16 ± 0.02 gen^-1^ in the evolved communities (*p* < 0.05 for a Student’s *t* test for all comparisons between ancestral and evolved communities, with Bonferroni correction applied for multiple testing). The respective changes in population densities for each species during these invasion experiments are shown in S4A Fig.

To determine if the higher fitness of the colicin-producing strain in the evolved communities as compared to the ancestral community was a result of either increased production of colicin or a higher susceptibility of the evolved *E*. *coli* to the toxin, we performed 2 separate experiments. First, we measured the total amount of protein present in the spent media in either the ancestral or 5 of the evolved co-culture communities in the presence of colicin-producing *E*. *coli*. This value was then normalized to the total number of colicin-producing *E*. *coli* (Materials and methods). Interestingly, the concentration of protein present in the spent media of the evolved communities was less than that observed in the ancestral communities, although the difference was not statistically significant (Student’s *t* test with Bonferroni correction applied for multiple comparisons, S5 Table and S5A Fig). To next determine if the evolved *E*. *coli* clones varied in their susceptibility towards colicin as compared to the ancestral *E*. *coli*, or that the presence of *S*. Typhimurium affects the susceptibility of *E*. *coli* towards colicin (for example, through secretion of specific proteases), we measured the size of the zone of inhibition produced by colicin ColE2 (the colicin secreted by ECOR 11) on a lawn of *E*. *coli* (ancestral and evolved), in the presence or absence of spent media from the cognate *S*. Typhimurium. We did not observe any difference between these zones of inhibitions (Student’s *t* test with Bonferroni correction applied for multiple comparisons; S6 Table and S5B and S5C Fig). Combined, our data shows that the higher fitness of the colicin-producing *E*. *coli* in the evolved community is not due to increased colicin production or due to increased susceptibility. This suggests that the lower selection coefficient of colicin-producing *E*. *coli* in the ancestral community might be due to differences in resource utilization between the ancestral and evolved communities.

### Eco-evolutionary dynamics in evolved communities result in a higher invasion rate of colicin-producing *E*. *coli*

**Fig 3:**
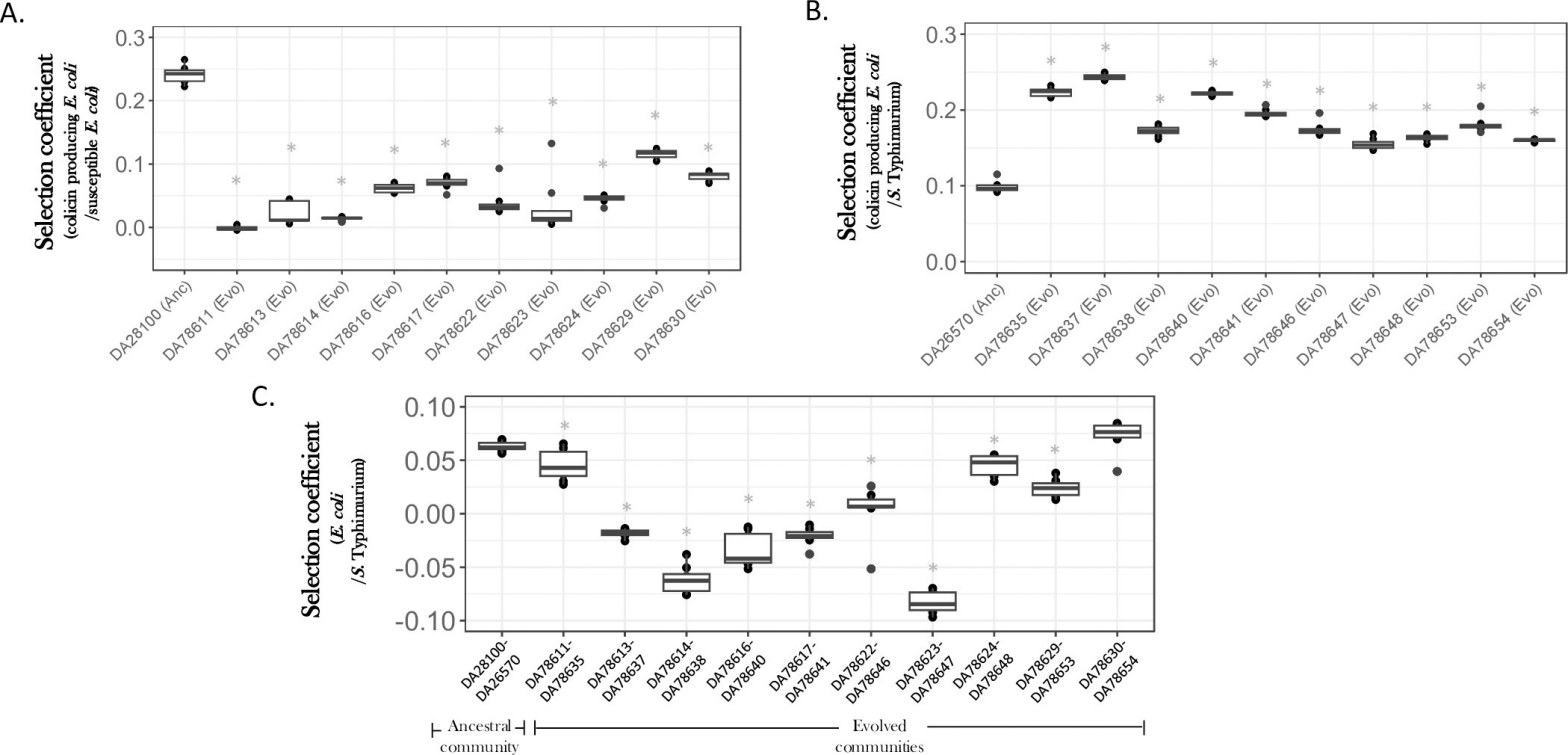
Two-way competitions reveal heightened interspecific competition and ecological specialization in the evolved communities. Two-way species interactions were determined by measuring selection coefficients for two-way competition experiments between (A) colicin-producing *E*. *coli* and susceptible *E*. *coli*, (B) colicin-producing *E*. *coli* and *S*. Typhimurium, and (C) cognate pairs of *E*. *coli* and *S*. Typhimurium. Eight replicates were used in each case and the line in the center represents the median value. In each case, ancestral clones are denoted as *Anc* and evolved clones are denoted as *Evo*. The X-axis represents the ancestral and evolved clones/communities and the Y-axis represents the selection coefficients showing the change in frequency of the first species with respect to the second species. A Student’s *t* test (with Bonferroni’s correction for multiple testing) was performed to determine statistically significant differences (denoted as * for *p* < 0.05) between the ancestral and each of the evolved states. The numbers on the X-axis represent the strain IDs for ancestral and evolved clones of *E*. *coli* and *S*. Typhimurium used in the study. The data underlying this figure can be found in S1 Data, in the sheet titled Fig 3.

To determine why the ancestral and evolved communities differed in their ability to resist invasion by a toxin-producing bacteria, we performed two-way competition experiments between the susceptible *E*. *coli*, S. Typhimurium, and colicin-producing *E*. *coli*. In the first of these experiments, we competed colicin-producing *E*. *coli* against susceptible *E*. *coli* (evolved and ancestral). These experiments showed that the colicin-producing *E*. *coli* outcompeted the ancestral susceptible *E*. *coli* at a higher rate as compared to the evolved susceptible *E*. *coli* strains (*p* < 0.05 for a Student’s *t* test for all comparisons between ancestral and evolved communities, with Bonferroni correction applied for multiple testing, Fig 3A and S7 Table). Thus, the selection coefficient for the rate of increase of the colicin-producing *E*. *coli* over ancestral susceptible *E*. *coli* was 0.24 ± 0.01 gen^-1^, while it varied between −0.001 ± 0.003 gen^-1^ to 0.11 ± 0.007 gen^-1^ for the evolved susceptible *E*. *coli*. We next competed the colicin-producing *E*. *coli* with *S*. Typhimurium (ancestral and evolved). We observed a higher rate of increase of colicin-producing *E*. *coli* against the evolved *S*. Typhimurium as compared to the ancestral *S*. Typhimurium (*p* < 0.05 for a Student’s *t* test for all comparisons between ancestral and evolved communities, with Bonferroni correction applied for multiple testing, Fig 3B and S7 Table). Thus, the selection coefficient for the rate of increase of the colicin-producing *E*. *coli* over ancestral *S*. Typhimurium was 0.1 ± 0.007 gen^-1^, while it varied between 0.15 ± 0.007 gen^-1^ to 0.24 ± 0.004 gen^-1^ for the evolved *S*. Typhimurium. Lastly, we competed the evolved *E*. *coli* strains against their cognate evolved *S*. Typhimurium clones to determine if the competitive differences between them were more or less similar to those observed for the ancestral *E*. *coli* and *S*. Typhimurium clones. These results showed that the evolved *S*. Typhimurium strains were competitively better than their respective evolved *E*. *coli* clones, as compared to the ancestral clones (*p* < 0.05 for a Student’s *t* test for all but one comparison between ancestral and evolved communities, with Bonferroni correction applied for multiple testing, Fig 3C and S7 Table). In other words, *S*. Typhimurium was doing better in the evolved communities as compared to the ancestral communities. The respective changes in population densities for each species during these two-way competition experiments are shown in S4B–S4D Fig.

These two-way competition results demonstrate that the evolved *E*. *coli* clones were fitter than the ancestral *E*. *coli* when competing against colicin-producing *E*. *coli*. This would imply that communities consisting of the evolved *E*. *coli* clones are less prone to invasion as compared to communities consisting of the ancestral *E*. *coli*. However, we observed the opposite. Furthermore, we observed that the evolved *S*. Typhimurium clones performed better in the evolved communities compared to the ancestral communities, despite having increased resource overlap with their cognate evolved *E*. *coli* clones compared to the ancestral clones (see previous results from spent medium assays). Thus, it is likely that the combination of increased resource competition between the evolved *E*. *coli* and evolved *S*. Typhimurium and the reduced resource competition between the evolved *S*. Typhimurium and the colicin-producing *E*. *coli* results in a quicker turn-over of the *E*. *coli* population in these evolved communities.

### Modeling the effect of interspecific competition on invasion resistance of the community using a three-species Lotka–Volterra model

To determine if competition models could capture the eco-evolutionary dynamics that were observed in our experiments, we used a Lotka–Volterra interspecific competition model that was extended to include the invasion dynamics by a toxin-producing *E*. *coli* (Materials and methods). Briefly, our model simulated how the invasion by a pathogenic strain (starting at a low frequency) depends upon the strength of interspecific competition and the state of ecological specialization of different members of the community. Thus, in these models, we varied the competition coefficients between all 3 interacting species, i.e., susceptible *E*. *coli*, colicin-producing *E*. *coli*, and *S*. Typhimurium. In line with our experimental observations, we adjusted the interspecific competition by modifying the competition coefficient a_ES_ (Materials and methods), which represents the effect *S*. Typhimurium has on the growth of susceptible *E*. *coli*. The strength of interspecific competition was then calculated by taking the ratio between the appropriate interspecific and intraspecific competition coefficients, which in our model are a_ES_/a_EE._ Similarly, ecological specialization was altered by adjusting the competition coefficient a_CS_ (Materials and methods), which represents the effect of *S*. Typhimurium on the growth of the colicin-producing *E*. *coli*. These adjustments mirrored our observations of the changing interaction strength between *S*. Typhimurium and colicin-producing *E*. *coli* in the ancestral and evolved communities.

**Fig 4:**
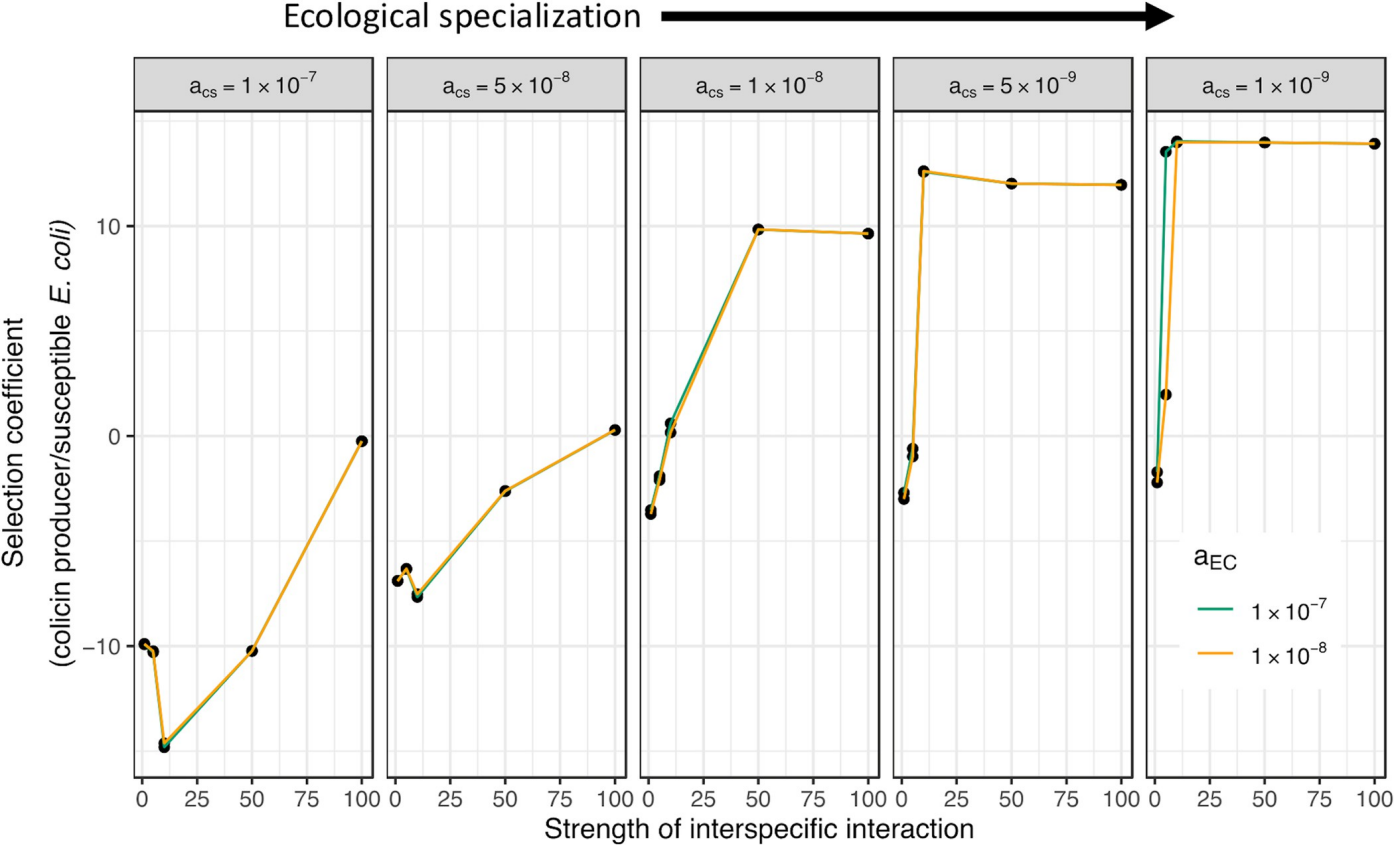
Lotka–Volterra interspecific competition model corroborates the link between interspecific competition and invasion success. Outcomes based on a three-species Lotka–Volterra interspecific competition model that was extended to include killing by toxin (Materials and methods). In this model, the strength of interspecies interactions and the degree of ecological specialization are varied by adjusting the two-way competition coefficients. Specifically, the effect of *S*. Typhimurium on the growth of susceptible *E*. *coli* (representing the strength of interspecies interaction) and colicin-producing *E*. *coli* (representing ecological specialization) is modified by changing the competition coefficient a_ES_ and a_CS_, respectively. The effect of colicin-producing *E*. *coli* on the growth of the susceptible *E*. *coli*, due to competition for resources, is varied by changing a_EC_ (represented as different colors in the figure)_._ X-axis represents the strength of interspecies interaction and is calculated as the ratio between a_ES_/a_EE_ (Materials and methods)_._ The selection coefficient represents the outcome of competition between the colicin-producing *E*. *coli* and susceptible *E*. *coli* in 3 species communities computed by numerically solving the model and is the measure of the invasion rate of the colicin-producing *E*. *coli*. A dependence of this invasion rate on the strength of interspecies interaction and ecological specialization is observed.

Overall, our model corroborates the findings from our experiments. That is, as the strength of interspecific competition between the different members of the community increases, it increases the invasion rate of the colicin-producing *E*. *coli* (Fig 4). Notably, these invasion rates ranged between the invader failing to establish itself (selection coefficient values below 0 in Fig 4) under low interspecific competition between the different members of the community, to a successful invasion (selection coefficient values above 0 in Fig 4) at higher values of strength of interspecific competition. Furthermore, in line with what we observed in our experiments, the invasion rate of the colicin-producing *E*. *coli* was much higher in communities where the toxin-resistant member of the community (*S*. Typhimurium in the synthetic communities used in this study) had undergone ecological specialization. Our model also predicted that as this state of ecological specialization increases, the effect of interspecific competition starts to plateau. Interestingly, we did not observe a widespread impact of the interaction strength between the colicin-producing *E*. *coli* and susceptible *E*. *coli* on the overall invasion rates (modeled by changing competition coefficient a_EC_).

Taken together, our model and experimental results show that changes in the strength of interspecific competition and ecological specialization in microbial communities can result in complex eco-evolutionary dynamics affecting the invasion resistance of the community.

## Discussion

**Fig 5:**
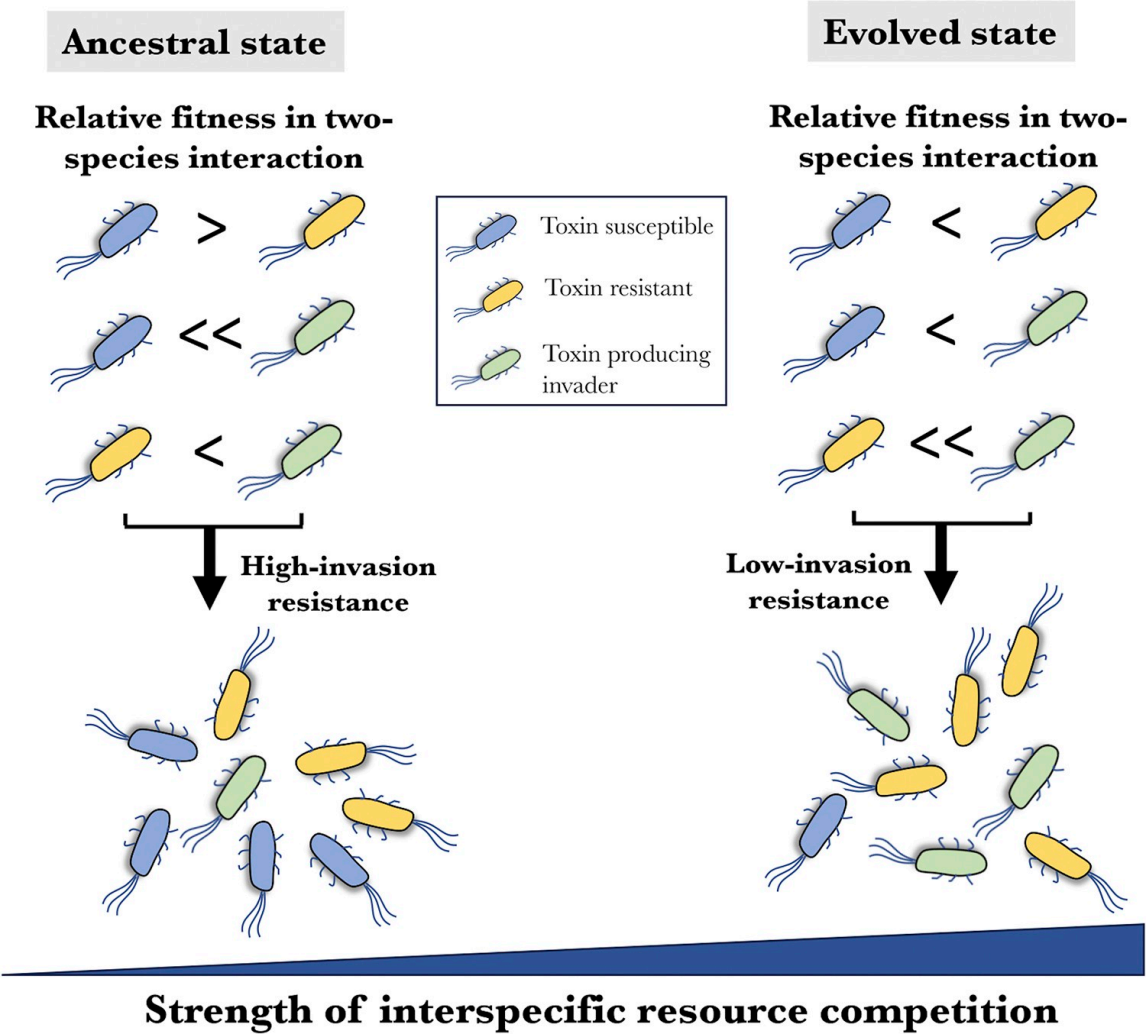
An increase in resource competition within a community results in a decrease in invasion resistance. A schematic representation of the interplay between resource competition and invasion resistance in microbial communities. As the strength of resource competition increases within the community, the resistance to a toxin-producing invader reduces. Furthermore, these invasion dynamics are not predictable based on two-way competition outcomes between the community members and the invader.

Several studies have demonstrated the importance of the host’s residential microbial community against pathogen invasion [26,27], but how evolutionary changes within a microbial community can affect its interaction with invading pathogens is lacking. Our study addresses this by determining how invasion by a colicin-producing *E*. *coli* is affected by adaptive changes in synthetic microbial communities. We used a colicin-producing *E*. *coli* in our experiments because colicin production is a widespread phenotype that can contribute to the invasion by several pathogens. Our results show that communities that have coexisted for longer periods and have accumulated adaptive changes are more prone to invasion by a colicin-producing bacterium (Fig 5). However, this is opposite to what is expected from existing ecological theory [20,28,29], which states that evolved communities demonstrate increased resistance to invasion. Our experimental data and mathematical simulations show that these observations are an outcome of a complex combination of evolutionary and ecological changes, based on increased resource consumption abilities and ecological specialization in these communities (Fig 5). Additionally, previous studies have not investigated the invasion due to a pathogen-specific phenotype (i.e., toxin production) in a microbial community but have focused on replacement through resource competition, which may explain the different results between our and previous studies.

Importantly, our study highlights the interplay between interspecies resource competition and invasion dynamics by a toxin-secreting bacterium. Although both of these occurrences are common in microbial communities across different ecosystems [30–32], the interplay between them is rarely investigated. Our study shows that the interaction between these features results in unintuitive dynamics. Thus, invasion resistance is lower when the community members that are not affected by the toxin evolve to have differential resource utilization abilities. In our experiments, this was observed when *S*. Typhimurium which was isolated from the evolved communities had increased in fitness compared to the ancestral *S*. Typhimurium, had a higher fitness against the cognate *E*. *coli* strains (as compared to the fitness of the ancestral *S*. Typhimurium against the ancestral *E*. *coli*), and demonstrated reduced competition against the invading colicin-producing *E*. *coli* (as compared to the ancestral *S*. Typhimurium). These observations could either be due to more efficient usage of resources or due to the use of different resources, both of which are likely outcomes in the complex growth media used in our study.

We further corroborated the dependence of invasion resistance on the strength of interspecies competition using a Lotka–Volterra interspecies competition model. In this model, we observed that invasion resistance to the colicin-producing *E*. *coli* decreases as the strength of interspecific competition between *S*. Typhimurium and *E*. *coli* increases. Furthermore, this effect becomes more pronounced with increasing ecological specialization of *S*. Typhimurium in these communities, which was achieved in our simulations by reducing the effect of *S*. Typhimurium on the growth of the invading colicin-producing *E*. *coli* (as was observed in our experiments). Interestingly, in our synthetic communities, interspecific competition (measured by the increased resource overlap and fitness measurements of *S*. Typhimurium with respect to susceptible *E*. *coli*) and ecologically specialization (measured by fitness measurements of colicin-producing *E*. *coli* with respect to *S*. Typhimurium) both increased over time. A possible explanation for these observations is that in these synthetic communities, the joint evolution of *E*. *coli* and *S*. Typhimurium results in both species becoming better adapted to utilizing common resources thus increasing interspecific competition. At the same time, the preferred usage of these common resources would result in other resources being available for any incoming invading species, thus resulting in the resident species being more ecologically specialized in relation to any incoming species. Although our results and model predictions are suggestive of these conclusions, more work is needed to understand the mechanisms and nature of ecological specialization of *E*. *coli* and *S*. Typhimurium in these communities.

Whole-genome sequencing revealed that all the mutations in the evolved *S*. Typhimurium clones were in genes that contributed to motility, i.e., *motA*, *cheR*, *fimH*, and *fljB*. The evolved clones harboring these mutations were also found to be more resource-specialized, as indicated by the reduced competition between the invading *E*. *coli* and *S*. Typhimurium strains (Fig 3B). Furthermore, evolved clones of *E*. *coli*, had mutations in genes that were involved in carbon metabolism and in cell-adhesion phenotypes. Although these results suggest the relevance of these phenotypes in contributing to the community dynamics we observe in our experiments, future experiments involving genetic reconstruction of these mutations in clean ancestral backgrounds are needed to conclusively establish a causal link between these mutations and resource utilization.

Interestingly, we also observed an opposite trend between invasion dynamics in two-species (toxin-susceptible and toxin-producing bacterium) versus three-species system (toxin-susceptible, toxin-producing bacterium, and toxin-resistant competitor). Thus, the ancestral *E*. *coli* was outcompeted by colicin-producing *E*. *coli* at a faster rate compared to the evolved *E*. *coli*. However, in a community consisting of ancestral *E*. *coli* and *S*. Typhimurium invasion by a colicin-producing *E*. *coli* was slower than in a community consisting of jointly evolved *E*. *coli* and S. Typhimurium clones. The differences between the two-species and three-species dynamics highlight the importance of understanding the mechanisms driving these interactions to make better predictions about invasion dynamics in microbial communities.

Connecting ecological theory with evolutionary outcomes for medically relevant phenotypes is an important step in making predictions about the evolution of these phenotypes. This study takes a step in this direction by using synthetic microbial communities, where invasion dynamics become predictable based on phenotypes of resource competition and toxin production. Disentangling the interactions between different members of the community further demonstrated why our observations are opposite to what one expects from the ecological theory of invasion, highlighting the need to understand the mechanisms that dictate these dynamics.

## Material and methods

### Bacterial strains and growth conditions

Three different bacterial species were used in this study which included *E*. *coli* K-12 MG1655, *S*. Typhimurium LT2, and ECOR 11 which is a natural isolate of *E*. *coli* that carries the colE2 plasmid [33]. In these experiments, we used *E*. *coli* K-12 MG1655 which carried a chromosomal copy of a gene encoding a yellow-fluorescent protein, and *S*. Typhimurium which carried a chromosomal copy of a gene encoding a blue-fluorescent protein. The nutrient-rich media used in this study was lysogeny broth (LB; 1 g Yeast extract, 1.5 g Tryptone, and 1.5 g Sodium chloride in 150 ml de-ionized water). All strains were grown at 37°C.

### Experimental evolution of synthetic microbial communities

Ten replicates of two-species synthetic communities were formed by mixing 1 μl each of overnight-grown cultures of *E*. *coli* K-12 MG1655 and *S*. Typhimurium in 200 μl of fresh LB media. This was allowed to grow for 24 h in microtiter plates at 37°C, after which 1 μl of the culture was transferred to 200 μl of fresh LB media, resulting in approximately 8 generations per transfer. This was continued for 55 transfers resulting in a total of approximately 440 generations. After every 10 transfers, the plates were stored at −80°C by adding 20 μl of DMSO to each well and mixing it thoroughly after which the plates were sealed with adhesive foils (VWR 60941–076). At the end of 55 transfers, single clones of *E*. *coli* and *S*. Typhimurium were isolated. After determining that these clones had an increased competitive fitness as compared to the ancestral strains (see below), cognate pairs were mixed to generate evolved communities.

### Growth assays, competitions, and invasion experiments

The increase in competitive abilities of the evolved strains was determined by either performing single species growth curves or by competition experiments against ancestral strains. The single species growth curves were obtained using a BioscreenC analyzer at OD_600_, with measurements taken every 4 min. Each growth curve measurement was initiated by making a 1:1,000 dilution of an overnight-grown culture in fresh media. The exponential growth rate was measured by fitting the OD_600_ values between 0.02 and 0.09 to an exponential growth equation *N* = N*e^rt^ using the KaleidaGraph software (v4.5.3), where r (min^-1^) represents the exponential growth rate. The stationary phase density was calculated using the R-package growthcurver [34]. Three biological replicates were used in each case. To determine the overlap of resource usage between *E*. *coli* K-12 MG1655 and *S*. Typhimurium, growth analysis in spent medium was performed. To prepare the spent medium, the ancestral and evolved clones of *E*. *coli* and *S*. Typhimurium were grown in 5 ml of LB individually. After the overnight growth, the cells were pelleted down by centrifugation, and the supernatant was filter sterilized using a 0.22 μm syringe filter. In cases where the spent medium had to be restocked with nutrients, a ~10-fold concentrated LB solution was used. (Although the concentrated stock solution was prepared starting with 10-fold amounts of different components of LB, a small amount of the components was left undissolved. These were later removed during filter sterilization.) Two replicates were used in each case. As before, the stationary phase density was calculated using the R-package growthcurver [34]. The stationary phase densities of the evolved clones of *E*. *coli* and *S*. Typhimurium are plotted relative to the values observed for the ancestral clones, with both being measured during the same experiment. It is important to note that since we are using a complex media with several carbon and nitrogen sources and where resource utilization would depend on the presence or absence of a second species, drawing conclusions about resource specialization from these spent media assays would be misleading. Thus, inferences about resource specialization were only made from competition experiments.

Competition experiments were performed by mixing 1 μl each of overnight grown cultures in 200 μl of fresh media. These experiments were performed for 2 days, with 1 μl of overnight-grown culture transferred to 200 μl of fresh media every day. The frequencies of different strains were measured by tagging the strains with genes that encoded different fluorescent proteins (yellow or blue, henceforth denoted as yfp and bfp, respectively). Thus, for the two-way competition experiments *E*. *coli* K-12 MG1655 was tagged with a yellow fluorescent protein (YFP), *S*. Typhimurium was tagged with the blue fluorescent protein, while the frequency of colicin-producing ECOR 11 *E*. *coli* was determined by counting the non-fluorescent cells. To assess the reliability of using fluorescently marked strains for competition experiments, 2 separate tests were conducted. First, to check for any background fluorescence in the colicin-producing non-fluorescent *E*. *coli*, fluorescence was measured in overnight cultures of this bacterium. No fluorescence signal was detected (S8 Table). Next, to determine if *E*. *coli* cells lysed by colicin would incorrectly be counted as non-fluorescent, we grew the YFP expressing *E*. *coli* for 24 h in 1 ml of LB that contained 50 μl of colicin-containing supernatant. This supernatant was prepared by growing an overnight culture of colicin-producing *E*. *coli*, removing the cells by centrifugation, followed by filtration through a 0.22 μm syringe filter. A comparison of growth curves of *E*. *coli* in LB media with and without the colicin-containing supernatant was done to confirm the effect of colicin-killing on growth (S6 Fig). Fluorescence measurements of the YFP-expressing *E*. *coli* grown in colicin-containing LB showed that no cells were categorized as non-fluorescent (S8 Table).

Competition experiments performed to determine the increase in fitness of the evolved clones of *E*. *coli* and S. Typhimurium also made use of fluorescently labeled ancestral strains. Thus, an ancestral strain of *E*. *coli* carrying the bfp was used to compete against the evolved clones of *E*. *coli* (that were labeled with yfp), while an ancestral strain of *S*. Typhimurium carrying a yfp was used to compete against the evolved clones of *S*. Typhimurium (that were labeled with bfp). For these competitions, control experiments were performed by competing ancestral strains carrying bfp against ancestral strains carrying yfp.

At the time of measurement of frequencies of differently labeled strains, 3 μl of cultures were diluted in 200 μl of phosphate-buffered saline and the fraction of differently labeled cells was determined using flow cytometry (MACSQuant VYB, Miltenyi Biotec). In all competition experiments, 8 biological replicates were used unless stated otherwise. The selection coefficient (s) was calculated by plotting natural logs of ratios of population sizes to time and by calculating the slope of the linearly regressed line such that s = [ln(X_t2_/ Y_t2_)—ln(X_t1_/ Y_t1_)]/(t_2_-t_1_) [35], where the population size of the respective strains is denoted by X and Y, and the 2 time-points where the population sizes were measured are denoted as t_2_ and t_1_. Since *E*. *coli* and *S*. Typhimurium coexist in LB, calculations of the increase in fitness of the evolved *E*. *coli* and *S*. Typhimurium clones with respect to one another were done by maintaining similar starting densities for the 2 species across the different competition experiments, and then measuring the rate of change of the respective population densities over ~15 generations. All selection coefficients were thus calculated before the different species/strains entered a steady state.

All invasion experiments performed utilized colicin E2-producing ECOR 11 *E*. *coli* strain as the invader. In these cases, an overnight-grown culture of ECOR 11 strain was diluted 1:100, and 1 μl of this diluted culture was mixed with either 1 μl of either *E*. *coli* or *S*. Typhimurium cultures (for invasion analysis in two-species systems), or 1 μl of each *E*. *coli* and *S*. Typhimurium (for invasion analysis in three-species systems) to start invasion experiments. The invasion experiments were carried out for 2 days and selection coefficients were calculated as described above. Eight replicates were used in each case. As stated previously, all selection coefficients were calculated before the different species/strains entered a steady state.

### Whole-genome sequence analysis

To determine the genetic basis of adaptation, 5 randomly chosen evolved clones were whole genome sequenced. DNA was extracted from 1 ml overnight cultures using the MasterPure Complete DNA & RNA Purification Kit (Epicentre) according to the manufacturer’s instructions. Illumina’s Nextera XT kit was used to make libraries (2 × 300) that were sequenced on Illumina’s Miseq platform. Samples were dual-indexed and pooled together. The average whole genome coverage per sample was approximately 30×. Analysis of the fastq files obtained from Miseq sequencing was performed using CLC genomics Workbench version 8 and was mapped to the reference genome of the ancestral *S*. Typhimurium LT2 strain or *E*. *coli* genome. SNP calling was also performed using this tool. To identify structural rearrangements, insertions, and deletions, Breseq (version 0.38.1) was used [36]. The fastq files used for whole genome analysis for the evolved clones can be found on NCBI’s sequence read archive (SRA, accession ID PRJNA1134789).

### Secreted protein determination and test for colicin sensitivity

To determine the amount the secreted proteins in the media, we first grew the respective three-species communities in 2 ml of LB. After 24 h of growth, the cells were pelleted by centrifugation at 13,000 rpm for 3 min and the supernatant was filter sterilized using a 0.22 μm syringe filter. Protein levels in this media were then measured using the Life Technologies Qubit protein assay kit (Q33211) and by following the manufacturer’s protocol. The total number of colicin-producing *E*. *coli* was determined by performing flow cytometry, as has been described above. Three replicates were used in each case.

Colicin sensitivity assay was performed by first growing colicin-producing *E*. *coli* ECOR 11 in 2 ml of LB. After 24 h of growth, the cells were pelleted down by centrifugation at 13,000 rpm for 3 min and the supernatant was filter sterilized using a 0.22 μm syringe filter. The filtrate was used as a source of colicin, and 100 μl of an overnight-grown test bacteria was then plated on an LB plate, after which 5 μl from the colicin-containing filtrate was spotted on the plate. Once the spot had dried, the plates were incubated for overnight growth at 37°C. The next day, the diameter of the zone of clearance was measured and used as a proxy for colicin sensitivity. To determine if *S*. Typhimurium’s secretome was somehow affecting colicin sensitivity, a filter-sterilized spent media from an overnight-grown *S*. Typhimurium culture was used. In this case, 100 μl of an overnight-grown test bacteria and 100 μl of the spent media were plated together, after which spotting was performed using the colicin-containing filtrate. Three replicates were used in each case.

### Three-species Lotka–Volterra model for interspecific competition and toxin-mediated killing

The three-species Lotka–Volterra model used in our study makes use of the following differential equations:

$$dEdt=r_{E}*E*(1‐a_{EE}*E‐a_{ES}*S‐a_{EC}*C)‐d*M*C*f$$ (1)

$$dSdt=r_{S}*S*(1‐a_{SS}*S‐a_{SE}*E‐a_{SC}*C)$$ (2)

$$dCdt=r_{C}*C*(1‐a_{CC}*C‐a_{CE}*E‐a_{CS}*S)‐d*M*C$$ (3)

where E, S, and C stand for the population size of *E*. *coli*, *S*. Typhimurium, and colicin-producing *E*. *coli*, respectively; r represents exponential growth rate (gen^-1^), a_ii_ represents intraspecific competition coefficients, i.e., the effect of a given species i on its growth rate; a_ij_ represents interspecific competition coefficients, i.e., the effect of species j on the growth rate of species i; d is the rate of cell-lysis involved in colicin secretion (gen^-1^), f is the amount of colicin secreted per cell per generation, and M is a factor that relates the dependence of population density to colicin production. To be consistent with our experiments, the different values of a_ii_ and a_ij_ used in these simulations are based on having a carrying capacity of approximately 10^9^ and using the rationale that carrying capacity equates inversely to competition coefficients [37]. In our model, the values of intraspecific competition coefficients were kept constant with a_EE_ = a_SS_ = a_CC_ = 10^−9^, while the strength of interspecific competition was varied by changing the values of a_ij_. Thus, the strength of interspecific competition between *E*. *coli* and *S*. Typhimurium was manipulated by changing the parameter a_ES_ (10^−7^, 5*10^−8^, 10^−8^, 5*10^−9^, 10^−9^) and was calculated as the ratio a_ES_/a_EE_ [37]. Ecological specialization was obtained by changing the parameter a_CS_ (10^−7^, 5*10^−8^, 10^−8^, 5*10^−9^, 10^−9^), i.e., by changing the effect of *S*. Typhimurium on the growth rate of colicin-producing *E*. *coli*. Similarly, the strength of interaction between *E*. *coli* and the colicin-producing *E*. *coli* was changed by changing the parameter a_EC_ (10^−7^, 10^−8^). Other interspecific competition coefficients were kept constant with a_SC_ = 10^−9^, a_SE_ = 10^−8^, and a_CE_ = 10^−8^. Parameter M was included since bacterial cells secrete colicins under nutrient-limiting conditions [38], which is in turn tied to the total bacterial density in the community. The value of M used is (E+C+S)/10^8^ since the carrying capacity of the community in this environment is approximately 10^9^. The values of r_E_, r_S_, and r_C_ are based on empirically determined exponential growth rates for *E*. *coli* and *S*. Typhimurium in LB, which are approximately 0.033 min^-1^ for both these species. For a generation time of approximately 20 min [39,40], this corresponds to a growth rate value of 0.66 gen^-1^. The value of d used throughout the simulations is 0.02 gen^-1^, i.e., at the onset of colicin production, 2% of the colicin-producing *E*. *coli* lyse open to secrete the colicins every generation, while the value of f is kept at 1,000. Based on previous work investigating the mechanism of action of colicins, we assume here that a single colicin molecule is sufficient to kill a single susceptible bacteria [22,41,42]. To get plottable values for selection coefficients, any value of the population size obtained during the simulation that was below 0 was changed to 0.0001. These sets of equations were numerically solved in R (Version 1.4.1717) [43] using the differential equation solver ode45 from the package deSolve [44].

### Statistical analysis

A Student’s *t* test was performed to determine the increase in fitness of the evolved clones by comparing it to fitness values observed in the appropriate control experiments (Fig 1), to determine differences in invasion resistance between the ancestral and evolved communities (Fig 2), to determine statistically significant differences for ancestral and evolved clones in the two-species competition (Fig 3), to determine differences in final ratios of the evolved clones as compared to the ancestral clones (S2 Fig), to determine differences in spent media assays between ancestral and evolved clones (S3 Fig), to determine if the concentration of secreted proteins was different between the evolved and ancestral communities (S5 Fig), as well as for determining differences in colicin susceptibility of evolved and ancestral *E*. *coli* (S5 Fig). A Bonferroni’s correction was applied to correct for multiple testing.

## Supporting information

S1 Fig *E*. *coli* and *S*. Typhimurium are involved in scramble type of resource competition in nutrient-rich lysogeny broth (LB). (A) Relative stationary phase density after 20 h of growth in either fresh LB, supernatant from overnight growth of the other species, or supernatant restocked with approximately 10×-concentrated LB is plotted for *E*. *coli* and *S*. Typhimurium. The values are made relative to the stationary phase density observed in fresh media. Two replicates were used in each case, and the error bars represent standard deviations. The data underlying this figure can be found in S1 Data, in the sheet titled S1A Fig. (B) Growth curves for *E*. *coli* and *S*. Typhimurium on fresh media, supernatant from overnight growth of the other species, or supernatant restocked with approximately 10×-concentrated LB. Two replicates are used in each case. The data underlying this figure can be found in S1 Data, in the sheet titled S1B Fig.
(TIF)

S2 Fig Cognate-evolved clones of *E*. *coli* and *S*. Typhimurium were grown together in LB for 5 cycles of growth to determine changes in species ratios between the ancestral and evolved communities. The Y-axis represents the average of *E*. *coli*: S. Typhimurium ratios relative to the ratios of the ancestral strains, after 5 days of growth. Eight replicates are used in each case, and error bars represents standard deviations. Statistically significant differences are determined by performing a Student’s *t* test, with Bonferroni’s correction applied for multiple testing (denoted as * for *p* < 0.05). The numbers on the X-axis represent the strain IDs for the cognate pairs of *E*. *coli* and *S*. Typhimurium used in the study. The data underlying this figure can be found in S1 Data, in the sheet titled S2 Fig.
(TIF)

S3 Fig Spent medium assays were performed to determine changes in resource usage abilities between ancestral and evolved communities of *E*. *coli* and *S*. Typhimurium. (A) Relative stationary phase densities (with respect to ancestral state values) of the ancestral and evolved *E*. *coli* on cell-free spent medium obtained from the ancestral and cognate-evolved *S*. Typhimurium, respectively. (B) Relative stationary phase densities (with respect to ancestral state values) of the ancestral and evolved *S*. Typhimurium on cell-free spent medium obtained from the ancestral and cognate-evolved *E*. *coli*, respectively. Two replicates are used for the ancestral and each of the evolved communities. Error bars represent standard deviations. Statistically significant differences are determined by performing a Student’s *t* test, with Bonferroni’s correction applied for multiple testing (denoted as * for *p* < 0.05). The numbers on the X-axis represent the strain IDs for ancestral and evolved clones of *E*. *coli* and *S*. Typhimurium used in the study. The data underlying this figure can be found in S1 Data, in the sheet titled S3 Fig (raw data and plotted).
(TIF)

S4 Fig Change in population densities for two- and three-species competition experiments. Log_10_ transformed values of population sizes of (A) susceptible *E*. *coli*, colicin-producing *E*. *coli*, and *S*. Typhimurium (three-species system), (B) susceptible *E*. *coli* and colicin-producing *E*. *coli* (two-species system), (C) colicin-producing *E*. *coli* and *S*. Typhimurium (two-species system), and (D) susceptible *E*. *coli* and *S*. Typhimurium (two-species system) grown together in LB for 15 generations. In each case, ancestral communities/clones are denoted as *Anc* and evolved communities/clones are denoted as *Evo*. Eight replicates were used in each case. The Y-axis shows log_10_ transformed values of population sizes/ml and X-axis shows time in generations. The numbers in the headers of each panel represent the strain IDs for *E*. *coli* and *S*. Typhimurium clones used in the study. The data underlying this figure can be found in S1 Data, in the sheet titled S4 Fig.
(TIFF)

S5 Fig (A) The concentration of colicin secreted per cell, measured by quantifying the total secreted protein and normalizing it to the total number of colicin-producing cells present, is plotted for the ancestral and evolved communities. The boxplot for evolved communities represents observation from 5 different communities, and the line in the center denotes the median value. Three replicates are used in each case. (B) Susceptibility to colicin ColE2 in nutrient-rich lysogeny broth, and (C) in the presence of supernatant from cognate *S*. Typhimurium is shown for ancestral and evolved *E*. *coli*. Five different evolved *E*. *coli* clones were used. Three replicates are used in each case. The data underlying this figure can be found in S1 Data, in the sheet titled S5 Fig.
(TIF)

S6 Fig Growth curves of yellow-fluorescent protein expressing *E*. *coli* in either LB (black) or in LB with 50 μl of colicin-containing supernatant (gray). X-axis represents time (mins) and Y-axis represents optical density measured at 600 nm. The data underlying this figure can be found in S1 Data, in the sheet titled S6 Fig.
(TIF)

S1 Table Increase in fitness of the evolved clones of *E*. *coli* and *S*. Typhimurium isolated after 55 cycles of growth as determined by performing competition experiments with respective ancestral strains. Four replicates were used in each case. Student’s *t* test was used to determine statistical significance by comparing values obtained for respective control experiments, and correction for multiple testing was done using Bonferroni’s correction method.
(DOCX)

S2 Table Stationary phase density and exponential growth rate of evolved isolates of *E*. *coli* and *S*. Typhimurium isolated after 55 cycles of growth. All values are normalized to the value for the respective ancestral strain. Three biological replicates are used in each case. Student’s *t* test was used to determine statistical significance, and correction for multiple testing was done using Bonferroni’s correction method.
(DOCX)

S3 Table Mutations observed in the evolved clones of *E*. *coli* and S. Typhimurium isolated after 55 cycles of growth. Mutations are shown as the type of mutation (i.e., IS element insertions (IS), deletions (Δ), or non-synonymous changes), position on the genes where the mutation is observed, and whether the mutation is in the coding region or is intergenic. Structural rearrangements are denoted as structural variant phase inversion.
(DOCX)

S4 Table Invasion resistance was determined by allowing colicin-producing *E*. *coli* (1% of total frequency) to invade ancestral and evolved communities of *E*. *coli* and *S*. Typhimurium. The success of invasion is determined by measuring selection coefficients. Eight biological replicates are used in each case. Student’s *t* test was used to determine statistically significant differences between the ancestral and evolved communities, with correction for multiple tests done using Bonferroni’s correction method.
(DOCX)

S5 Table Secreted protein concentrations per unit of colicin-producing bacterium for ancestral and evolved communities. All values are normalized to the values of the ancestral community. Three biological replicates are used in each case. Student’s *t* test was used to determine statistical significance, and correction for multiple testing was done using Bonferroni’s correction method.
(DOCX)

S6 Table Susceptibility to colicin ColE2 was measured for ancestral and evolved *E*. *coli*. Colicin susceptibility was also determined in the presence of supernatant obtained from cognate *S*. Typhimurium clones. Student’s *t* test was used to determine statistical significance, and correction for multiple testing was done using Bonferroni’s correction method. The data underlying this figure can be found in S1 Data, in the sheets titled “colicin_susceptibility” and “colicin_suscep._Sal_spentmedia.”
(DOCX)

S7 Table Selection coefficients were determined for two-way competition experiments between *E*. *coli* (ancestral and evolved), *S*. Typhimurium (ancestral and evolved), and colicin-producing *E*. *coli*. Student’s *t* test was used to determine statistically significant differences between the ancestral and evolved communities, with correction for multiple tests done using Bonferroni’s correction method.
(DOCX)

S8 Table The reliability of using fluorescently marked bacteria in competition experiments was assessed for non-fluorescent colicin-producing *E*. *coli* and for lysed, yellow-fluorescent protein-coding colicin-sensitive *E*. *coli*.
(DOCX)

S1 Data Raw data files for all the experiments used in the study. (XLS)

S2 Data The R script (including annotations) used to simulate the Lotka–Volterra interspecies competition model. (R)
