# Supplementary figures and images for: The strength of interspecies interaction in a microbial community determines its susceptibility to invasion
Source: PLoS Biol. 2024 Nov 7;22(11):e3002889. doi: 10.1371/journal.pbio.3002889 (PMC11575764; doi:10.1371/journal.pbio.3002889)

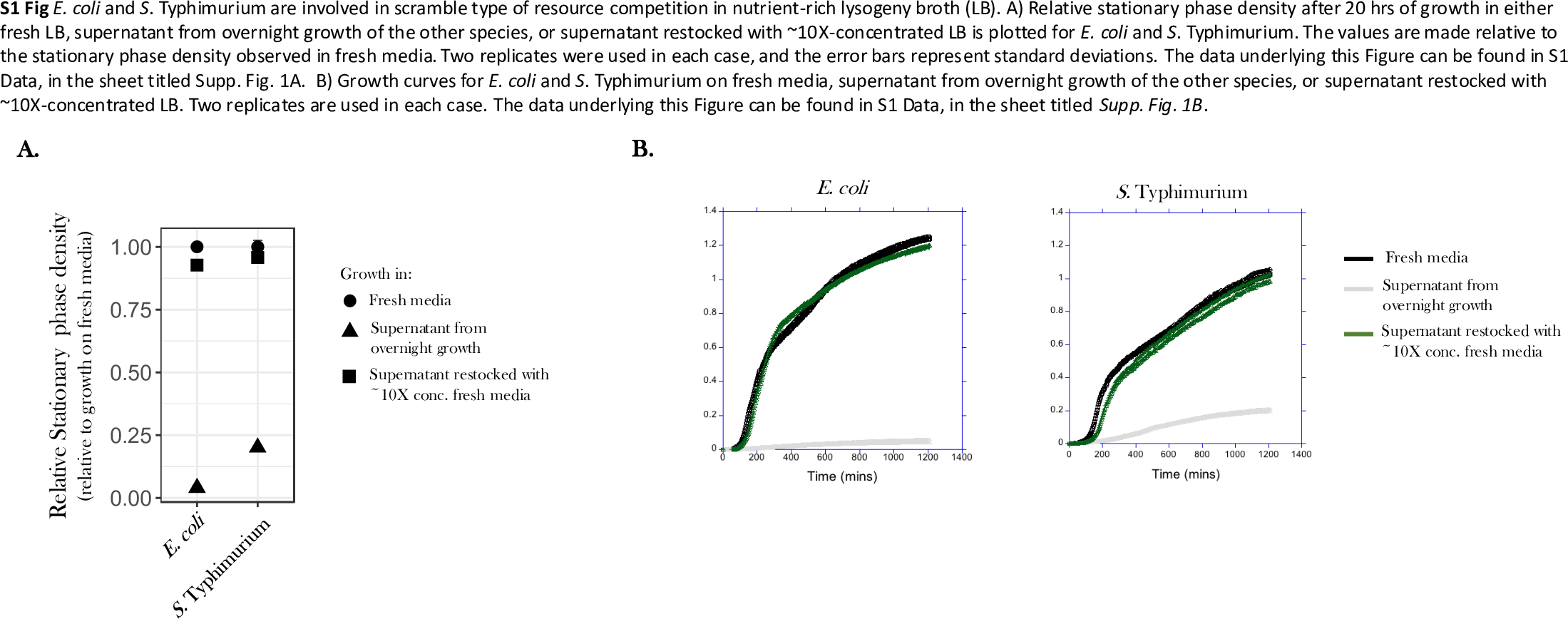

Supplement: S1 Fig — (A) Relative stationary phase density after 20 h of growth in either fresh LB, supernatant from overnight growth of the other species, or supernatant restocked with approximately 10×-concentrated LB is plotted for E. coli and S. Typhimurium. The values are made relative to the stationary phase density observed in fresh media. Two replicates were used in each case, and the error bars represent standard deviations. The data underlying this figure can be found in S1 Data, in the sheet titled S1A Fig. (B) Growth curves for E. coli and S. Typhimurium on fresh media, supernatant from overnight growth of the other species, or supernatant restocked with approximately 10×-concentrated LB. Two replicates are used in each case. The data underlying this figure can be found in S1 Data, in the sheet titled S1B Fig. (TIF) [file pbio.3002889.s001.tif]

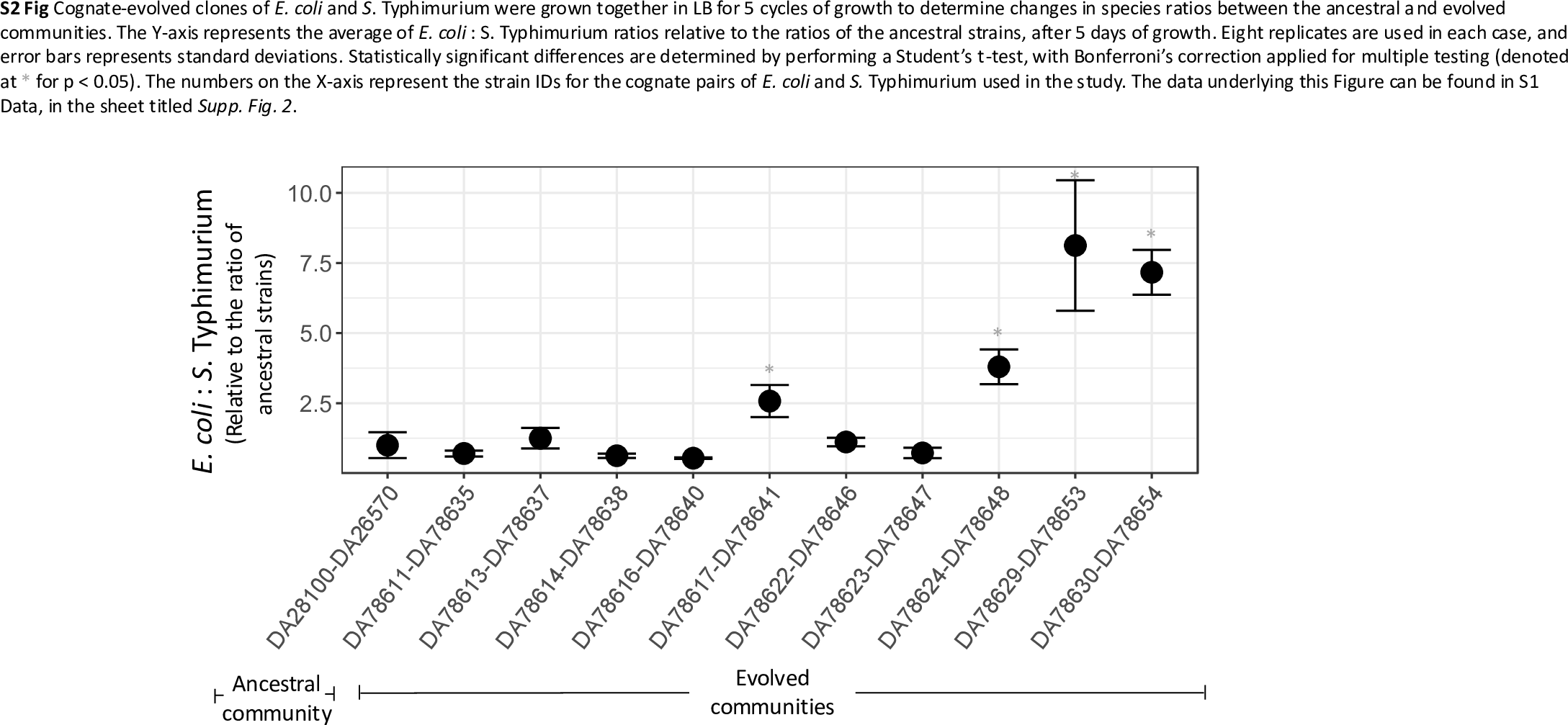

Supplement: S2 Fig — The Y-axis represents the average of E. coli: S. Typhimurium ratios relative to the ratios of the ancestral strains, after 5 days of growth. Eight replicates are used in each case, and error bars represents standard deviations. Statistically significant differences are determined by performing a Student’s t test, with Bonferroni’s correction applied for multiple testing (denoted as * for p < 0.05). The numbers on the X-axis represent the strain IDs for the cognate pairs of E. coli and S. Typhimurium used in the study. The data underlying this figure can be found in S1 Data, in the sheet titled S2 Fig. (TIF) [file pbio.3002889.s002.tif]

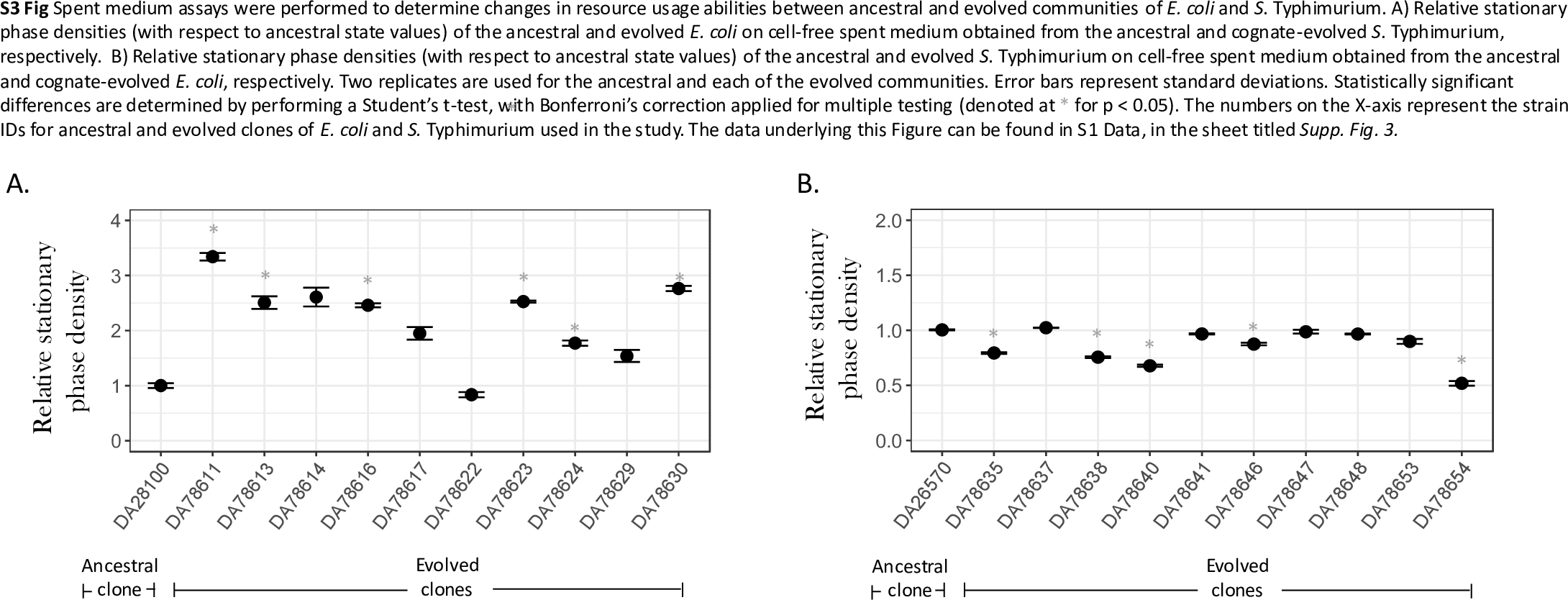

Supplement: S3 Fig — (A) Relative stationary phase densities (with respect to ancestral state values) of the ancestral and evolved E. coli on cell-free spent medium obtained from the ancestral and cognate-evolved S. Typhimurium, respectively. (B) Relative stationary phase densities (with respect to ancestral state values) of the ancestral and evolved S. Typhimurium on cell-free spent medium obtained from the ancestral and cognate-evolved E. coli, respectively. Two replicates are used for the ancestral and each of the evolved communities. Error bars represent standard deviations. Statistically significant differences are determined by performing a Student’s t test, with Bonferroni’s correction applied for multiple testing (denoted as * for p < 0.05). The numbers on the X-axis represent the strain IDs for ancestral and evolved clones of E. coli and S. Typhimurium used in the study. The data underlying this figure can be found in S1 Data, in the sheet titled S3 Fig (raw data and plotted). (TIF) [file pbio.3002889.s003.tif]

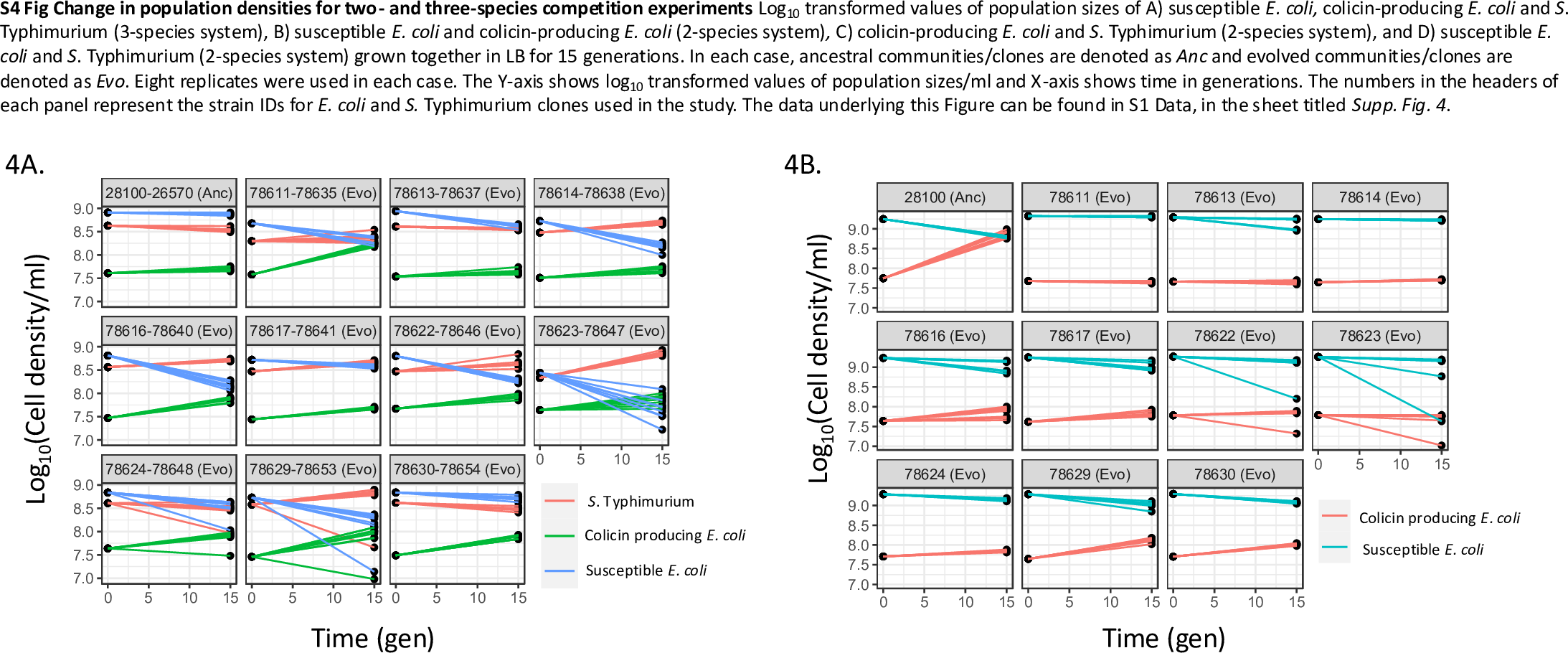

Supplement: S4 Fig — Log10 transformed values of population sizes of (A) susceptible E. coli, colicin-producing E. coli, and S. Typhimurium (three-species system), (B) susceptible E. coli and colicin-producing E. coli (two-species system), (C) colicin-producing E. coli and S. Typhimurium (two-species system), and (D) susceptible E. coli and S. Typhimurium (two-species system) grown together in LB for 15 generations. In each case, ancestral communities/clones are denoted as Anc and evolved communities/clones are denoted as Evo. Eight replicates were used in each case. The Y-axis shows log10 transformed values of population sizes/ml and X-axis shows time in generations. The numbers in the headers of each panel represent the strain IDs for E. coli and S. Typhimurium clones used in the study. The data underlying this figure can be found in S1 Data, in the sheet titled S4 Fig. (TIFF) [file pbio.3002889.s004.tiff]

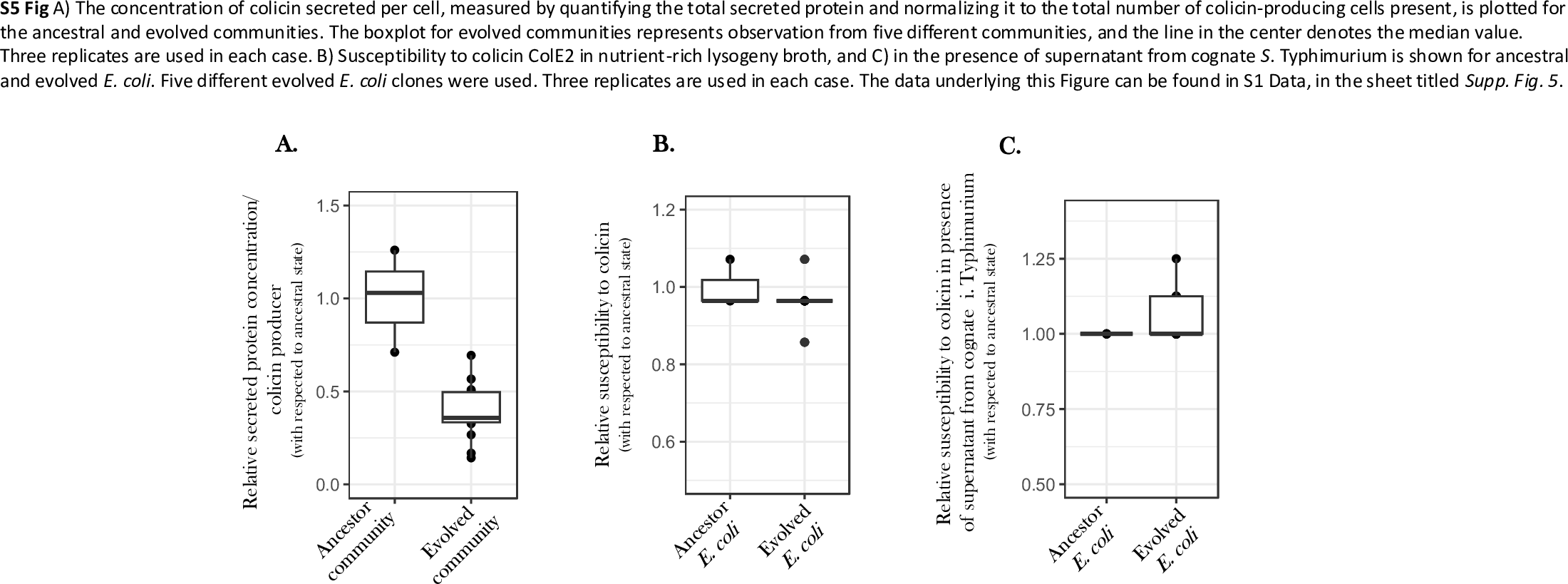

Supplement: S5 Fig — (A) The concentration of colicin secreted per cell, measured by quantifying the total secreted protein and normalizing it to the total number of colicin-producing cells present, is plotted for the ancestral and evolved communities. The boxplot for evolved communities represents observation from 5 different communities, and the line in the center denotes the median value. Three replicates are used in each case. (B) Susceptibility to colicin ColE2 in nutrient-rich lysogeny broth, and (C) in the presence of supernatant from cognate S. Typhimurium is shown for ancestral and evolved E. coli. Five different evolved E. coli clones were used. Three replicates are used in each case. The data underlying this figure can be found in S1 Data, in the sheet titled S5 Fig. (TIF) [file pbio.3002889.s005.tif]

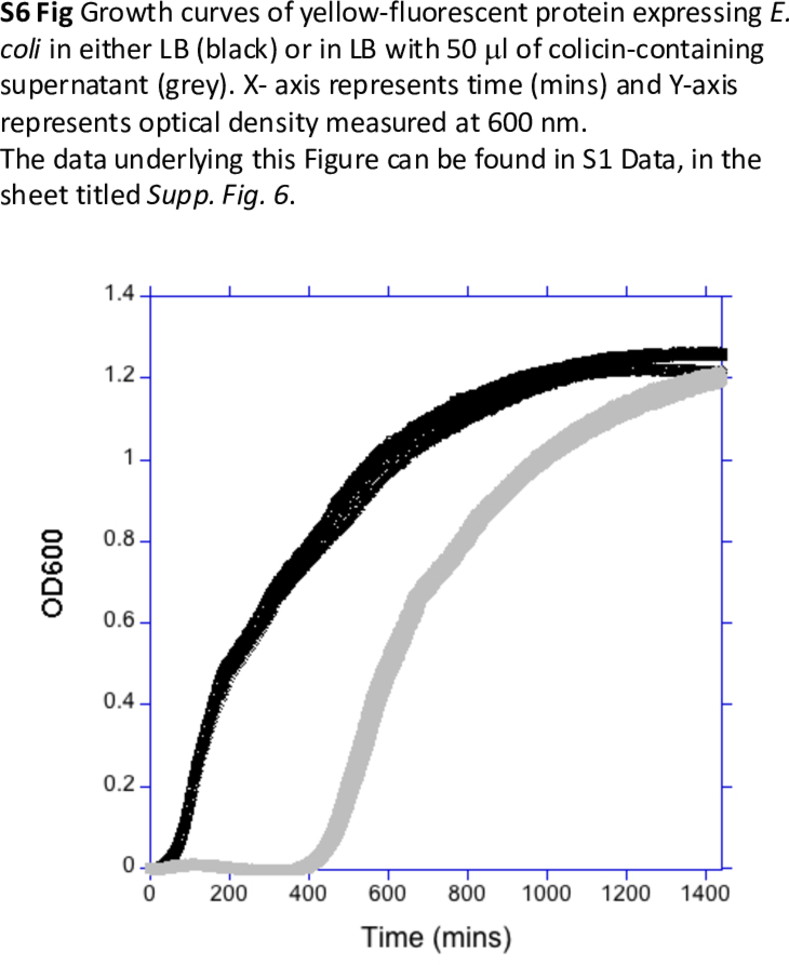

Supplement: S6 Fig — X-axis represents time (mins) and Y-axis represents optical density measured at 600 nm. The data underlying this figure can be found in S1 Data, in the sheet titled S6 Fig. (TIF) [file pbio.3002889.s006.tif]
