# Supplementary material for: The strength of interspecies interaction in a microbial community determines its susceptibility to invasion
Source: PLoS Biol. 2024 Nov 7;22(11):e3002889. doi: 10.1371/journal.pbio.3002889 (PMC11575764; doi:10.1371/journal.pbio.3002889)
Supplement: S1 Table — Increase in fitness of the evolved clones of E. coli and S. Typhimurium isolated after 55 cycles of growth as determined by performing competition experiments with respective ancestral strains. Four replicates were used in each case. Student’s t test was used to determine statistical significance by comparing values obtained for respective control experiments, and correction for multiple testing was done using Bonferroni’s correction method. (DOCX) [file pbio.3002889.s007.docx]

| **Species** | **Strain type** | **Strain_ID** | **Average** ± **Std. Dev.** | **adj. p-value (Bonferroni's correction)** |
| --- | --- | --- | --- | --- |
| ***E. coli*** | Ancestral (control exp.) | DA28100 | 0.0320 ± 0.0016 | - |
| ***E. coli*** | Evolved | DA78611 | 0.2096 ± 0.0016 | 1.7E-21 |
| ***E. coli*** | Evolved | DA78613 | 0.1452 ± 0.0185 | 1.0E-15 |
| ***E. coli*** | Evolved | DA78614 | 0.1604 ± 0.0095 | 9.4E-19 |
| ***E. coli*** | Evolved | DA78616 | 0.1368 ± 0.0081 | 8.9E-18 |
| ***E. coli*** | Evolved | DA78617 | 0.1592 ± 0.0037 | 7.3E-20 |
| ***E. coli*** | Evolved | DA78622 | 0.1934 ± 0.0118 | 1.1E-19 |
| ***E. coli*** | Evolved | DA78623 | 0.1869 ± 0.0074 | 1.8E-20 |
| ***E. coli*** | Evolved | DA78624 | 0.1773 ± 0.0141 | 2.0E-18 |
| ***E. coli*** | Evolved | DA78629 | 0.1679 ± 0.0063 | 7.5E-20 |
| ***E. coli*** | Evolved | DA78630 | 0.1811 ± 0.0087 | 6.4E-20 |
| ***S.* Typhimurium** | Ancestral  (control exp.) | DA26570 | -0.0278 ± 0.0026 | - |
| ***S.* Typhimurium** | Evolved | DA78635 | 0.0722 ± 0.0119 | 2.3E-09 |
| ***S.* Typhimurium** | Evolved | DA78637 | 0.0784 ± 0.0058 | 7.9E-11 |
| ***S.* Typhimurium** | Evolved | DA78638 | 0.0965 ± 0.0069 | 1.2E-12 |
| ***S.* Typhimurium** | Evolved | DA78640 | 0.0988 ± 0.0110 | 1.9E-12 |
| ***S.* Typhimurium** | Evolved | DA78641 | 0.0644 ± 0.0060 | 8.4E-09 |
| ***S.* Typhimurium** | Evolved | DA78646 | 0.0624 ± 0.0060 | 1.8E-08 |
| ***S.* Typhimurium** | Evolved | DA78647 | 0.3669 ± 0.0152 | 5.9E-22 |
| ***S.* Typhimurium** | Evolved | DA78648 | 0.0687 ± 0.0151 | 1.9E-08 |
| ***S.* Typhimurium** | Evolved | DA78653 | 0.3621 ± 0.0107 | 1.7E-22 |
| ***S.* Typhimurium** | Evolved | DA78654 | 0.3652 ± 0.0122 | 2.4E-22 |

**S1 Table**. Increase in fitness of the evolved clones of *E. coli* and *S*. Typhimurium isolated after 55 cycles of growth as determined by performing competition experiments with respective ancestral strains. Four replicates were used in each case. Student’s t-test was used to determine statistical significance by comparing values obtained for respective control experiments, and correction for multiple testing was done using Bonferroni’s correction method.
