# Supplementary material for: The strength of interspecies interaction in a microbial community determines its susceptibility to invasion
Source: PLoS Biol. 2024 Nov 7;22(11):e3002889. doi: 10.1371/journal.pbio.3002889 (PMC11575764; doi:10.1371/journal.pbio.3002889)
Supplement: S2 Table — Stationary phase density and exponential growth rate of evolved isolates of E. coli and S. Typhimurium isolated after 55 cycles of growth. All values are normalized to the value for the respective ancestral strain. Three biological replicates are used in each case. Student’s t test was used to determine statistical significance, and correction for multiple testing was done using Bonferroni’s correction method. (DOCX) [file pbio.3002889.s008.docx]

**S2 Table**. Stationary phase density and exponential growth rate of evolved isolates of *E. coli* and *S*. Typhimurium isolated after 55 cycles of growth. All values are normalized to the value for the respective ancestral strain. Three biological replicates are used in each case. Student’s t-test was used to determine statistical significance, and correction for multiple testing was done using Bonferroni’s correction method. The data underlying this Figure can be found in S1 Data, in the sheet titled ‘Exp.rate and Stat. den.’.

| **Species** | **Strain type** | **Strain_ID** | **Stationary phase density** | | **Exponential growth rate** | |
| --- | --- | --- | --- | --- | --- | --- |
|  |  |  | **Average** ± **Std. Dev.** | **Adjusted p-value** | **Average** ± **Std. Dev.** | **Adjusted p-value** |
| *E. coli* | Ancestral | DA28100 | 1.00 ± 0.013 |  | 1.00 ± 0.10 |  |
| *E. coli* | Evolved | DA78611 | 1.10 ± 0.01 | 0.003 | 0.79 ± 0.03 | 0.275 |
| *E. coli* | Evolved | DA78613 | 1.06 ± 0.01 | 0.024 | 0.78 ± 0.05 | 0.333 |
| *E. coli* | Evolved | DA78614 | 1.01 ± 0.01 | 1.000 | 0.74 ± 0.08 | 0.286 |
| *E. coli* | Evolved | DA78616 | 0.99 ± 0.03 | 1.000 | 0.65 ± 0.03 | 0.049 |
| *E. coli* | Evolved | DA78617 | 1.04 ± 0.03 | 0.783 | 0.84 ± 0.06 | 0.844 |
| *E. coli* | Evolved | DA78622 | 1.05 ± 0.00 | 0.031 | 0.86 ± 0.06 | 1.000 |
| *E. coli* | Evolved | DA78623 | 1.06 ± 0.01 | 0.057 | 0.75 ± 0.01 | 0.154 |
| *E. coli* | Evolved | DA78624 | 1.11 ± 0.01 | 0.003 | 0.94 ± 0.05 | 1.000 |
| *E. coli* | Evolved | DA78629 | 1.13 ± 0.01 | 0.001 | 0.88 ± 0.08 | 1.000 |
| *E. coli* | Evolved | DA78630 | 1.03 ± 0.01 | 0.365 | 0.76 ± 0.05 | 0.245 |
| *S.* Typhimurium | Ancestral | DA26570 | 1.00 ± 0.01 |  | 1.00 ± 0.02 |  |
| *S.* Typhimurium | Evolved | DA78635 | 1.19 ± 0.03 | 0.011 | 1.03 ± 0.01 | 1.575 |
| *S.* Typhimurium | Evolved | DA78637 | 0.80 ± 0.01 | 0.000 | 1.01 ± 0.02 | 1.000 |
| *S.* Typhimurium | Evolved | DA78638 | 1.09 ± 0.07 | 0.858 | 1.06 ± 0.02 | 0.388 |
| *S.* Typhimurium | Evolved | DA78640 | 0.85 ± 0.02 | 0.005 | 0.99 ± 0.04 | 1.000 |
| *S.* Typhimurium | Evolved | DA78641 | 1.08 ± 0.05 | 0.496 | 0.98 ± 0.06 | 1.000 |
| *S.* Typhimurium | Evolved | DA78646 | 0.89 ± 0.04 | 0.109 | 1.07 ± 0.01 | 0.081 |
| *S.* Typhimurium | Evolved | DA78647 | 0.98 ± 0.20 | 1.000 | 0.99 ± 0.04 | 1.000 |
| *S.* Typhimurium | Evolved | DA78648 | 1.11 ± 0.09 | 1.000 | 0.98 ± 0.06 | 1.000 |
| *S.* Typhimurium | Evolved | DA78653 | 1.01 ± 0.18 | 1.000 | 1.00 ± 0.02 | 1.000 |
| *S.* Typhimurium | Evolved | DA78654 | 1.16 ± 0.03 | 0.012 | 0.93 ± 0.04 | 0.454 |
