# Supplementary material for: The strength of interspecies interaction in a microbial community determines its susceptibility to invasion
Source: PLoS Biol. 2024 Nov 7;22(11):e3002889. doi: 10.1371/journal.pbio.3002889 (PMC11575764; doi:10.1371/journal.pbio.3002889)
Supplement: S3 Table — Mutations observed in the evolved clones of E. coli and S. Typhimurium isolated after 55 cycles of growth. Mutations are shown as the type of mutation (i.e., IS element insertions (IS), deletions (Δ), or non-synonymous changes), position on the genes where the mutation is observed, and whether the mutation is in the coding region or is intergenic. Structural rearrangements are denoted as structural variant phase inversion. (DOCX) [file pbio.3002889.s009.docx]

**S3 Table.** Mutations observed in the evolved clones of *E. coli* and S. Typhimurium isolated after 55 cycles of growth. Mutations are shown as the type of mutation (i.e IS element insertions (IS), deletions (Δ), or non-synonymous changes), position on the genes where the mutation is observed, and whether the mutation is in the coding region or is intergenic. Structural rearrangements are denoted as structural variant phase inversion.

| **Species** | **Strain_ID** | **Gene** | **Mutation** | **Description of gene function** |
| --- | --- | --- | --- | --- |
| *E. coli* | DA78611 | *mglA* | IS2 (+) +5 bp; coding (339‑343/1521nt) | D‑galactose/methyl‑galactoside ABC transporter ATP binding subunit |
| *E. coli* | DA78611 | *fimB-* *fimE* | IS5 (–) +4 bp; intergenic (+434/‑41) | Type 1 fimbriae regulatory protein FimB/regulator for *fimA* |
| *E. coli* | DA78613 | *ptsG* | IS5 (+) +4 bp; coding (20‑23/1434 nt) | glucose‑specific PTS enzyme IIBC component |
| *E. coli* | DA78614 | *pgaA* | L407I | partially deacetylated poly‑beta‑1,6‑N‑acetyl‑D‑glucosamine export outer membrane porin |
| *E. coli* | DA78616 | *yehF-yehI* | IS186 (–) +7 bp :: Δ3 bp; intergenic (+696/‑2279) | PF05406 family protein YehF/DUF4132 domain‑containing protein YehI |
| *E. coli* | DA78616 | *fimE* | IS5 (–) +4 bp; coding (272‑275/597 nt) | regulator for fimbriae protein coding gene *fimA* |
| *E. coli* | DA78617 | *insA5-* *uspC* | IS5 (–) +4 bp; intergenic (‑271/‑264) | IS1 family protein InsA/universal stress protein C |
| *E. coli* | DA78617 | *ycfH-ptsG* | Δ1 bp :: IS186 (+) +6 bp :: Δ1 bp; intergenic (+231/‑59) | putative metal‑dependent hydrolase YcfH/glucose‑specific PTS enzyme IIBC component |
| *S*. Typhimurium | DA78635 | *fljB* | Structural variant_phase inversion | Flagellin protein |
| *S*. Typhimurium | DA78637 | *cheR* | Δ9 bp in frame deletion at 2014445 to 2014453 | chemotaxis protein (methyltransferase) |
| *S*. Typhimurium | DA78638 | *motA- flhC* | A→C; intergenic (‑7/+118) | proton conductor component of motor/regulator of flagellar biosynthesis |
| *S*. Typhimurium | DA78638 | *fljB* | Structural variant phase inversion | Flagellin protein |
| *S*. Typhimurium | DA78639 | *fljB* | Structural variant phase inversion | Flagellin protein |
| *S*. Typhimurium | DA78640 | *fljB* | Structural variant phase inversion | Flagellin protein |
| *S*. Typhimurium | DA78640 | *fimH* | G61A | minor fimbrial subunit |
| *S*. Typhimurium | DA78640 | *motA* | K236R | proton conductor component of flagellar motor complex |
