# Supplementary material for: The strength of interspecies interaction in a microbial community determines its susceptibility to invasion
Source: PLoS Biol. 2024 Nov 7;22(11):e3002889. doi: 10.1371/journal.pbio.3002889 (PMC11575764; doi:10.1371/journal.pbio.3002889)
Supplement: S4 Table — Invasion resistance was determined by allowing colicin-producing E. coli (1% of total frequency) to invade ancestral and evolved communities of E. coli and S. Typhimurium. The success of invasion is determined by measuring selection coefficients. Eight biological replicates are used in each case. Student’s t test was used to determine statistically significant differences between the ancestral and evolved communities, with correction for multiple tests done using Bonferroni’s correction method. (DOCX) [file pbio.3002889.s010.docx]

| ***E. coli* ID** | ***S*. Typhimurium ID** | **Community**  **Type** | **Selection coefficients (invading colicin producer/*E. coli*)** | **adj. p-value (Bonferroni's correction)** |
| --- | --- | --- | --- | --- |
| DA28100 | DA26570 | Ancestor | 0.018 ± 0.008 | - |
| DA78611 | DA78635 | Evolved | 0.15 ± 0.015 | 1.9047E-11 |
| DA78613 | DA78637 | Evolved | 0.064 ± 0.012 | 4.1713E-06 |
| DA78614 | DA78638 | Evolved | 0.11 ± 0.017 | 1.394E-08 |
| DA78616 | DA78640 | Evolved | 0.158 ± 0.018 | 1.2422E-10 |
| DA78617 | DA78641 | Evolved | 0.0588 ± 0.006 | 2.9658E-07 |
| DA78622 | DA78646 | Evolved | 0.121 ± 0.009 | 1.9391E-11 |
| DA78623 | DA78647 | Evolved | 0.154 ± 0.055 | 7.0598E-05 |
| DA78624 | DA78648 | Evolved | 0.086 ± 0.011 | 9.0968E-09 |
| DA78629 | DA78653 | Evolved | 0.152 ± 0.018 | 2.6542E-10 |
| DA78630 | DA78654 | Evolved | 0.08 ± 0.009 | 1.9833E-08 |

**S4 Table.** Invasion resistance was determined by allowing colicin-producing *E. coli* (1% of total frequency) to invade ancestral and evolved communities of *E. coli* and *S*. Typhimurium. The success of invasion is determined by measuring selection coefficients. Eight biological replicates are used in each case. Student’s t-test was used to determine statistically significant differences between the ancestral and evolved communities, with correction for multiple tests done using Bonferroni’s correction method.
