# Supplementary material for: The strength of interspecies interaction in a microbial community determines its susceptibility to invasion
Source: PLoS Biol. 2024 Nov 7;22(11):e3002889. doi: 10.1371/journal.pbio.3002889 (PMC11575764; doi:10.1371/journal.pbio.3002889)
Supplement: S5 Table — Secreted protein concentrations per unit of colicin-producing bacterium for ancestral and evolved communities. All values are normalized to the values of the ancestral community. Three biological replicates are used in each case. Student’s t test was used to determine statistical significance, and correction for multiple testing was done using Bonferroni’s correction method. (DOCX) [file pbio.3002889.s011.docx]

| ***E. coli* ID** | ***S*. Typhimurium ID** | **Community Type** | **Relative secretd protein conc./cell** | **Stdev** |
| --- | --- | --- | --- | --- |
| DA28100 | DA26570 | Ancestor | 1.00 | 0.27 |
| DA78611 | DA78635 | Evolved | 0.39 | 0.10 |
| DA78614 | DA78638 | Evolved | 0.45 | 0.08 |
| DA78616 | DA78640 | Evolved | 0.55 | 0.15 |
| DA78617 | DA78641 | Evolved | 0.32 | 0.05 |
| DA78623 | DA78647 | Evolved | 0.27 | 0.20 |

**S5 Table.** Secreted protein concentrations per unit of colicin-producing bacterium for ancestral and evolved communities. All values are normalized to the values of the ancestral community. Three biological replicates are used in each case. Student’s t-test was used to determine statistical significance, and correction for multiple testing was done using Bonferroni’s correction method.
