# Supplementary material for: The strength of interspecies interaction in a microbial community determines its susceptibility to invasion
Source: PLoS Biol. 2024 Nov 7;22(11):e3002889. doi: 10.1371/journal.pbio.3002889 (PMC11575764; doi:10.1371/journal.pbio.3002889)
Supplement: S6 Table — Susceptibility to colicin ColE2 was measured for ancestral and evolved E. coli. Colicin susceptibility was also determined in the presence of supernatant obtained from cognate S. Typhimurium clones. Student’s t test was used to determine statistical significance, and correction for multiple testing was done using Bonferroni’s correction method. The data underlying this figure can be found in S1 Data, in the sheets titled “colicin_susceptibility” and “colicin_suscep._Sal_spentmedia.” (DOCX) [file pbio.3002889.s012.docx]

| ***E. coli* ID** | **Strain type** | **Diameter of zone of no growth/inhibition (cm)** | | | | | |
| --- | --- | --- | --- | --- | --- | --- | --- |
|  |  | **-** | | | **with supernatant from cognate**  ***S*. Typhimurium** | | |
|  |  | **Replicate 1** | **Replicate 2** | **Replicate 3** | **Replicate 1** | **Replicate 2** | **Replicate 3** |
| DA28100 | Ancestral | 0.9 | 1 | 0.9 | 0.8 | 0.8 | 0.8 |
| DA78611 | Evolved | 0.9 | 0.9 | 0.9 | 0.9 | 1 | 0.8 |
| DA78614 | Evolved | 0.9 | 0.9 | 0.9 | 0.9 | 0.8 | 0.8 |
| DA78616 | Evolved | 1 | 0.9 | 0.9 | 0.8 | 0.9 | 0.8 |
| DA78617 | Evolved | 0.9 | 0.8 | 0.9 | 0.8 | 0.8 | 0.9 |
| DA78623 | Evolved | 0.9 | 0.9 | 0.9 | 0.9 | 0.8 | 0.8 |

**S6 Table.** Susceptibility to colicin ColE2 was measured for ancestral and evolved *E. coli.* Colicin susceptibility was also determined in the presence of supernatant obtained from cognate *S*. Typhimurium clones. Student’s t-test was used to determine statistical significance, and correction for multiple testing was done using Bonferroni’s correction method. The data underlying this Figure can be found in S1 Data, in the sheets titled ‘colicin_susceptibility’ and ‘colicin_suscep._Sal_spentmedia’.
