# Supplementary material for: The strength of interspecies interaction in a microbial community determines its susceptibility to invasion
Source: PLoS Biol. 2024 Nov 7;22(11):e3002889. doi: 10.1371/journal.pbio.3002889 (PMC11575764; doi:10.1371/journal.pbio.3002889)
Supplement: S7 Table — Selection coefficients were determined for two-way competition experiments between E. coli (ancestral and evolved), S. Typhimurium (ancestral and evolved), and colicin-producing E. coli. Student’s t test was used to determine statistically significant differences between the ancestral and evolved communities, with correction for multiple tests done using Bonferroni’s correction method. (DOCX) [file pbio.3002889.s013.docx]

| ***E. coli* ID** | ***S*. Typhimurium ID** | **Type** | **Selection coefficients for two-way competitions between** | | | | | |
| --- | --- | --- | --- | --- | --- | --- | --- | --- |
|  |  |  | ***E. coli* vs colicin producer** | | **colicin producer vs *S*. Tyhphimurium** | | ***E. coli* vs *S*. Tyhphimurium** | |
|  |  |  | **Avg ± SD** | **adj. p-value** | **Avg ± SD** | **adj. p-value** | **Avg ± SD** | **adj. p-value** |
| WT_ancestral | WT_ancestral | Ancestor | 0.24 ± 0.01 |  | 0.10 ± 0.01 |  | 0.06 ± 0.00 |  |
| DA78611 | DA78635 | Evolved | -0.001 ± 0.003 | 4.6E-16 | 0.22 ± 0.00 | 1.9E-14 | 0.05 ± 0.01 | 0.05 |
| DA78613 | DA78637 | Evolved | 0.02 ± 0.02 | 1E-12 | 0.20 ± 0.01 | 6.4E-16 | -0.02 ± 0.00 | 7.8E-15 |
| DA78614 | DA78638 | Evolved | 0.01 ± 0.002 | 9.8E-16 | 0.22 ± 0.01 | 5.8E-11 | -0.06 ± 0.01 | 3.5E-12 |
| DA78616 | DA78640 | Evolved | 0.06 ± 0.01 | 1E-13 | 0.24 ± 0.00 | 2.4E-15 | -0.03 ± 0.02 | 1.3E-09 |
| DA78617 | DA78641 | Evolved | 0.07 ± 0.01 | 4.3E-13 | 0.17 ± 0.01 | 4.7E-13 | -0.02 ± 0.01 | 3.4E-12 |
| DA78622 | DA78646 | Evolved | 0.04 ± 0.02 | 3E-11 | 0.17 ± 0.01 | 3.9E-10 | 0.00 ± 0.02 | 5.6E-05 |
| DA78623 | DA78647 | Evolved | 0.03 ± 0.04 | 3.2E-08 | 0.16 ± 0.01 | 4.2E-09 | -0.08 ± 0.01 | 1.4E-14 |
| DA78624 | DA78648 | Evolved | 0.04 ± 0.01 | 2.4E-14 | 0.16 ± 0.00 | 5.2E-11 | 0.05 ± 0.01 | 0.004 |
| DA78629 | DA78653 | Evolved | 0.12 ± 0.01 | 1.6E-11 | 0.18 ± 0.01 | 3.3E-10 | 0.02 ± 0.01 | 1.2E-07 |
| DA78630 | DA78654 | Evolved | 0.08 ± 0.01 | 4.4E-13 | 0.16 ± 0.00 | 2.4E-11 | 0.07 ± 0.01 | 0.78 |

**S7 Table.** Selection coefficients were determined for two-way competition experiments between *E. coli* (ancestral and evolved), *S*. Typhimurium (ancestral and evolved), and colicin-producing *E. coli*. Student’s t-test was used to determine statistically significant differences between the ancestral and evolved communities, with correction for multiple tests done using Bonferroni’s correction method.
