# Supplementary material for: The strength of interspecies interaction in a microbial community determines its susceptibility to invasion
Source: PLoS Biol. 2024 Nov 7;22(11):e3002889. doi: 10.1371/journal.pbio.3002889 (PMC11575764; doi:10.1371/journal.pbio.3002889)
Supplement: S8 Table — The reliability of using fluorescently marked bacteria in competition experiments was assessed for non-fluorescent colicin-producing E. coli and for lysed, yellow-fluorescent protein-coding colicin-sensitive E. coli. (DOCX) [file pbio.3002889.s014.docx]

| ***E. coli* type** | **Proportion of cells giving** | |
| --- | --- | --- |
|  | **Flourescent signal** | **Non-flourescent signal** |
| Colicin-producing E. coli | 0 | 100 |
| Yellow flourescent protein coding *E. coli* treated with colicin | 100 | 0 |

**S8 Table.** The reliability of using fluorescently marked bacteria in competition experiments was assessed for non-fluorescent colicin-producing *E. coli* and for lysed, yellow-fluorescent protein-coding colicin-sensitive *E. coli.*
